# A cadaver-based biomechanical model of acetabulum reaming for surgical virtual reality training simulators

**DOI:** 10.1038/s41598-020-71499-5

**Published:** 2020-09-03

**Authors:** Luigi Pelliccia, Mario Lorenz, Christoph-E. Heyde, Maximilian Kaluschke, Philipp Klimant, Sebastian Knopp, Stefan Schleifenbaum, Christian Rotsch, René Weller, Michael Werner, Gabriel Zachmann, Dirk Zajonz, Niels Hammer

**Affiliations:** 1 Professorship Machine Tool Design and Forming Technology, Professorship Factory Planning and Factory Operation, Chemnitz University of Technology, Reichenhainer Straße 70, 09126 Chemnitz, Germany; 2 Department of Orthopedics, Trauma and Plastic Surgery, University Hospital Leipzig, Liebigstraße 20, 04103 Leipzig, Germany; 3 Department of Computer Graphics and Virtual Reality, University of Bremen, Bibliothekstraße 5, 28359 Bremen, Germany; 4 Fraunhofer Institute for Machine Tools and Forming Technology IWU, Nöthnitzer Straße 44, 01187 Dresden, Germany; 5 Department of Macroscopic and Clinical Anatomy, Medical University of Graz, Harrachgasse 21, 8010 Graz, Austria

**Keywords:** Computational models, Cartilage

## Abstract

Total hip arthroplasty (THA) is a highly successful surgical procedure, but complications remain, including aseptic loosening, early dislocation and misalignment. These may partly be related to lacking training opportunities for novices or those performing THA less frequently. A standardized training setting with realistic haptic feedback for THA does not exist to date. Virtual Reality (VR) may help establish THA training scenarios under standardized settings, morphology and material properties. This work summarizes the development and acquisition of mechanical properties on hip reaming, resulting in a tissue-based material model of the acetabulum for force feedback VR hip reaming simulators. With the given forces and torques occurring during the reaming, Cubic Hermite Spline interpolation seemed the most suitable approach to represent the nonlinear force–displacement behavior of the acetabular tissues over Cubic Splines. Further, Cubic Hermite Splines allowed for a rapid force feedback computation below the 1 ms hallmark. The Cubic Hermite Spline material model was implemented using a three-dimensional-sphere packing model. The resulting forces were delivered via a human–machine-interaction certified KUKA iiwa robotic arm used as a force feedback device. Consequently, this novel approach presents a concept to obtain mechanical data from high-force surgical interventions as baseline data for material models and biomechanical considerations; this will allow THA surgeons to train with a variety of machining hardness levels of acetabula for haptic VR acetabulum reaming.

## Introduction

Total hip arthroplasty (THA) is considered one of the most successful procedures in orthopedic surgery^1^ with more than 200,000 cases *per annum* performed in Germany^2^ and a predicted number of 500,000 per annum in 2020 in the United States alone^3^. In spite of THA being a highly successful procedure, a number of complications related to this surgery exist, including aseptic loosening, early dislocation following THA, and misalignment. Some of these complications may be related to the surgical techniques deployed for THA.

To date, there is a lack of training opportunities for the various steps involved in THA in a standardized setting and without putting the patient at risk by the less experienced trainee. Human cadaveric tissues may here serve as an ideal model for trainees to gain experience, especially when supported by experienced senior colleagues. However, these scenarios are missing a standardized setting with predictable structure and haptics of the hip, which would be helpful to get a general appreciation for basic techniques and forces applied to the human system while at the same time having the opportunity to train the same intervention with exactly the same conditions.

Further to this, the approach using cadavers has limitations regarding the availability, and potential changes induced by the post mortem interval or the chemicals used for anatomical fixation^4–6^. Virtual Reality (VR) provides new opportunities to establish highly standardized training scenarios with realistic haptic behavior for THA surgery. Interventions simulated with VR can be trained potentially for an indefinite number of times. VR has already been successfully established in arthroscopy or laparoscopic surgery^7–9^. However, to date, no VR-based simulation, which includes realistic haptic feedback, has been developed for THA. Existing commercial VR simulators for THA are not providing a realistic haptic feedback due to the lack of haptic devices, which are capable of delivering the necessary high forces. In case of commercial VR-based simulators, it is also often unclear if the underlying material models used for the haptic rendering are basing on empirical biomechanical data or the feeling of surgeons involved in their development process. Whilst a lot can be learned from solely visual training simulation, especially for the acquisition of surgical skills, a realistic haptic simulation is at least as important; surgeons rely a lot on their haptic feeling during surgeries. Therefore, one of the main challenges for the development of a haptic VR-based THA simulation environment, is to provide a realistic force-feedback (haptic-feedback) to the user. A number of steps in THA need to be modeled, including the (1) surgical approach, (2) incision of the joint and removal of the femoral head, (3) reaming of the acetabulum and (4) femur, as well as (5) implanting the prosthetic components and (6) wound closure. Both haptic and visual properties are of fundamental interest to develop THA simulators in VR. A feasible approach to provide a realistic haptic feedback is to develop a material model to be used for predicting the interactional forces between the surgical tools and the different tissues.

In recent years, a plethora of research has been conducted aiming to identify the material properties of the acetabulum by using both cadaveric experiments^10,11^ and Finite Element Analysis (FEA)^12^. These studies’ results could potentially be used to build a simulation model of the acetabulum, e.g., based on FEA, and simulate its biomechanical interaction with the reamer. However, the simulator is required to deliver a real-time force feedback to the user updated with a high frequency. Whilst there is no absolute threshold for the update rate to deliver a reliable and stiff haptic behavior it is shown that faster update rates are leading to stiffer behavior^13,14^. This represents one of the main requirements for realistic surgical VR simulators. As a rule-of-thumb-criteria, an update rate of 1 kHz is considered to provide reliable and stiff haptic behavior^15^, although even at lower update rates, comparable results could be achievable^16^. We decided to set 1 kHz as the requirement for the force feedback calculation speed. Such performance would usually not be feasible using the FEA due to the computational load it requires.

Addressing these issues, this given study measured the direct interactional forces between the reamer and the acetabulum, determined an analytical representation of force-torque as a function of reamer displacement, and implemented this material model in the simulation environment. This study presents the very first step towards developing a surgery simulator capable of delivering biomechanical-based force feedback. Based on the mechanical data, the objective was to develop a VR-based surgery simulator capable of computing the haptic feedback in 1 ms. The resulting data was implemented into a human–machine-interaction certified robotic arm as a force feedback device, equally capable of reacting in the 1 ms timeframe as part of the surgical VR training and as a safety feature for the user.

To achieve this goal, the measured data were interpolated to provide a mathematical description of force-torque as a function of reamer displacement. Two interpolation techniques were implemented to provide an effective mathematical approximation of the data, optimizing computational time. The workup will be described and discussed in detail, including the mechanical testing setup and a self-developed fixture for holding the reamer and the acetabulum in a standardized manner, the reaming itself, as well as the material data and related material models.

## Materials and methods

Mechanical tests were conducted on cadaveric acetabular specimens to assess their biomechanical behavior under conditions similar to hip surgery. The tissues were obtained in accordance with the Saxonian Death and Funeral Act (version 2014) and University of Otago ethics approval (H17/20), and the experimental protocols were approved by these given institutions. While alive, the body donors gave their informed consent to the donation of their tissues for research purposes. Twenty-four cadaveric acetabula were retrieved from human cadavers (16 females, 8 males; mean age 87 ± 8 years, age range 74–102 years).

### Sample preparation

Surrounding soft tissues, including the hip muscles, were grossly removed from the hips before the ilio-, ischio- and pubofemoral ligaments were exposed^17^. Care was taken to transect these ligaments as distally as possible before dislocating the femoral heads. The capsular ligaments and the teres ligament of the hip joint were then removed carefully at the level of the acetabular labrum. Following this, the innominate bone with the intact acetabulum was trimmed for all specimens with dimensions fitting into the customized support device, while assuring that the cartilage, the entire subchondral bone lamella and sufficient bone stock measuring at least 15-mm in all directions was still adjacent to the specimen. It was then embedded in ceramic-reinforced polyurethane resin (RenShape solutions, Huntsman International LLC, Salt Lake City, USA) in the support device, with the resin having just enough viscosity to allow thorough anchoring of the specimen with no excessive resin penetration. The labral rim of the acetabulum was positioned using a custom-developed three-dimensional (3D) positioning device the support was mounted on. Care was taken to align the labral rim perpendicular to the axis of rotation of the reamer. Prior to the mechanical tests, the tissues were rinsed in isotonic 0.9 mass% saline and kept moist throughout the mechanical experiments.

### Experimental setup

**Figure 1:**
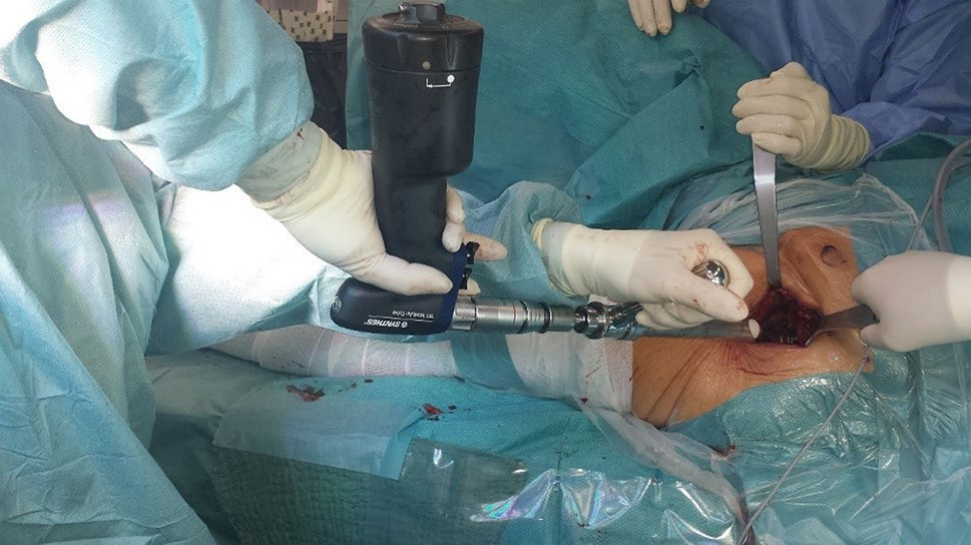
Reaming of the acetabulum during the hip surgery. The operating surgeon (on the left) uses a reaming tool to remove cartilage from the acetabulum of a left sided hip joint. Retractors hold open the surgical site, facilitated by two assisting surgeons (hands on the right).

**Figure 2:**
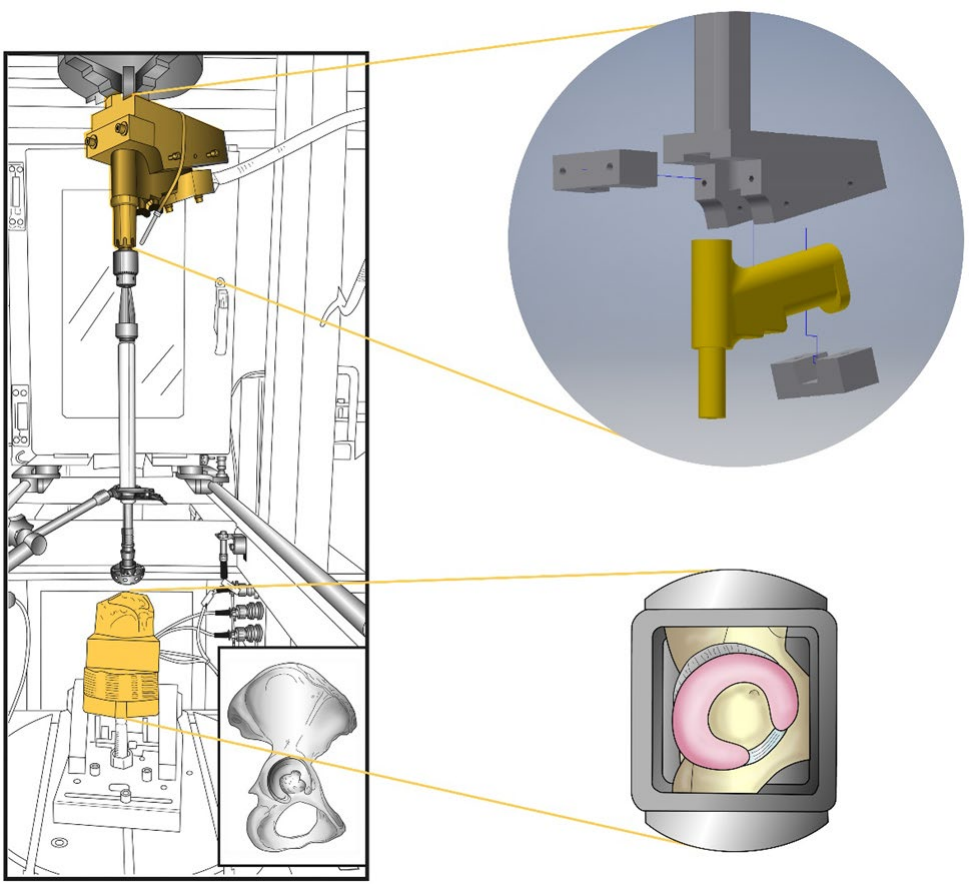
Experimental setup (left) showing the mounted specimen in a bi-axial testing machine and the reamer mounted above. Reaming tools of matching sizes were used for each of the specimens. The insert shows a right sided innominate bone. The explosion view on the right top shows the hand piece of the reaming tool and a customized fixture to attach it to the bi-axial testing machine. Bottom right shows the top view of the acetabular region of a right sided innominate bone mounted in a mold prior to the reaming experiments. The cartilage is depicted in pink.

Both the experimental setup and protocol were developed to replicate the real surgery conditions as closely as possible. Starting from observation of real surgery (see Fig. 1), an experimental setup was built (see Fig. 2). The reamer was connected to a biaxial material testing machine (DYNA-MESS, Aachen, Germany, 10 kN and 200 Nm certified measure range) to determine forces and torques along the direction of the reaming (see Fig. 2). For the connection of the reamer with the test machine and the embedding of the acetabula, dedicated fixators were CAD designed and manufactured from aluminum (see Fig. 2).

Forces and torques were measured along the direction of the reaming tool axis between the reamer and the acetabulum under both static (no reaming of acetabulum cartilage) and dynamic (reaming of acetabulum cartilage) conditions. The torques were caused by the friction that arose between the reaming tool and the acetabulum when the cartilage was removed. Thus, during the static tests, no torque arose. Three test series were conducted on each sample—one static test followed by two dynamic tests. All tests started from a position at which the contact force between the switched off reamer and the sample was 20 N. The test cycle began after that the reamer was switched into operating mode. For the tests, cadaveric tissue samples were numbered according to the test sequence followed in the experimental protocol (e.g., see sample 22 representing the 22nd test conducted). The angle of the reamer rotation was kept perpendicular relative to the equator of the acetabulum for standardization purposes in order to allow for baseline data acquisition.

During the **static tests**, the compression force was measured when the reamer was pushed into the acetabulum with a constant feed rate of 0.07 mm/s. The test stopped when (1) the force reached 800 N or (2) the tool displacement reached 20 mm. When performing **dynamic tests**, the reamer was operating to remove cartilage from the acetabulum in two consecutive trials with two different reaming tool sizes (labeled Dynamic 1 and Dynamic 2, respectively). The reaming tool for the test was chosen to match the diameter of the acetabulum, which was measured using a precision caliper. For the first trial, once the diameter of the acetabulum was measured (e.g. 51.2 mm), the next larger reamer with the rounded size was used (e.g. 52 mm), and for the second trial, the subsequently 2-mm larger reaming tool was chosen (e.g. 54 mm). The samples for the dynamic tests were further split into subgroups using two different feed rates, 0.01 mm/s and 0.03 mm/s. Dynamic tests were stopped if (1) the friction between the reamer and the sample caused the reamer to discontinue rotating, or (2) the tool displacement in the reaming direction exceeded 20 mm. These numbers and conditions assured that the cartilage was removed entirely in all the specimens.

This test setup measured displacement, force and torque (for dynamic data) in the reaming direction, with a sample time of 0.01 s. Thus, the test outcomes are displacement, force and torque (for dynamic data) vectors sampled at 0.01 s. As the measurements were conducted along one axis only, just one of the three vector components is meaningful, while the other two components are zero.

## Calculation

### Data filtering, normalization and interpolation

Force and torque data coming from dynamic tests were filtered by determining the appropriate cut-off frequencies for each dataset by means of a Fourier analysis, in order to build customized low-pass filters for each test, and to remove unwanted oscillations. Force data coming from the static tests did not require filtering as the reaming tool was turned off.

Force and torque data coming from both static and dynamic tests were then normalized with reference to the maximum vertical displacement for each test set, to compare the different sample responses. For determining an analytical description of forces and torques, piecewise interpolation was implemented using both Cubic Splines and Cubic Hermite Splines to approximate the filtered and normalized data. Using spline interpolation to approximate the measured curves largely reduces the computational and data storage requirements of the material model. The appropriate number of interpolation nodes within the displacement interval was automatically determined by means of a customized algorithm, considering the second order derivative of the force/torque diagram.

Specifically, a threshold for the second derivative was set and the points in which the derivative was above the threshold were selected. The aim of the algorithm was to obtain larger numbers of nodes in the interval in which the slope variations were higher. To determine the appropriate number of nodes to be used for the interpolation, a desired value of the maximum norm of the approximation error (absolute error value) between the measured data and the interpolation curve was set, defined as follows:

$${e}_{a}=\frac{v-{v}_{approx}}{v}\times 100,$$

where $v$ and ${v}_{approx}$ were the exact and the approximated values, respectively.

It is worth noticing that the given definition of error only aimed at providing a numerical value to compare the behavior of the interpolation curves, in addition to the visual comparison of the resulting graphs. For a desired value of the maximum error, starting from the maximum value of the second derivative, the threshold of the second derivative is recursively decreased by a defined sample step, and the algorithm recursively determines the number of nodes, calculating the related maximum norm of the approximation error. The calculation stopped when the maximum error was less or equal to the desired error value. The value of displacement at which the desired maximum error can also be determined by the algorithm. Lagrange interpolation schemes represented the easiest solution to the interpolation problem of N points. It defined an interpolation polynomial of minimum degree (N-1) which fitted the N points to be interpolated. The generic expression for the polynomial p of degree N-1 was $p\left(x\right)= {a}_{0}+{a}_{1}x+{a}_{2}{x}^{2}+\dots +{a}_{N-1}{x}^{N-1}$ whose values in each node are equal to the desired values in that node, i.e., the data values *f*_*i*_ at the nodes. This resulted in a set of N equations.

$$p\left({x}_{i}\right)={f}_{i} , i=0,\ldots,N,$$

that allowed the determination of the N unknown polynomial coefficients. The polynomial degree directly depended on the number of nodes. Specifically, the degree of the polynomial increased with the number of nodes considered. When a large number of nodes was interpolated, the resulting polynomial heavily oscillated in the nodes close to the boundary of the interpolation intervals (so called Runge phenomenon)^18^. To provide a computationally effective model avoiding Runge’s phenomenon, the data were processed using a piecewise interpolation, based on a third-order Spline^19,20^. The piecewise interpolation approach using both Cubic Splines and Cubic Hermite Splines allowed working with polynomials of fixed degree, independent from the number of nodes considered, avoiding Runge’s phenomenon. Once the appropriate number N of nodes was determined, the displacement interval was divided in N-1 sub-intervals $$\left[\begin{array}{cc}{d}_{i}& {d}_{i+1}\end{array}\right]$$, defined by each pair of nodes. The points *d*_*i*_ and *d*_*i*+*1*_ were called knots and the related Spline in each of the interval was defined as follows.

1 $${s}_{i}\left(d\right)={a}_{i}{\left(d-{d}_{i}\right)}^{3}+{b}_{i}{(d-{d}_{i})}^{2}+{c}_{i}(d-{d}_{i})+{d}_{i}.$$

The coefficients of the polynomial in each interval, for the Cubic Spline, were determined by setting the values of the Spline equal to the desired values *f*_*i*_ in each node, as well as the continuity of the Spline and its first and second derivative at the nodes in order to achieve a steady connection between the spline segments (piecewise-spline curve is C^2^ in knot). The aforementioned conditions lead to the following system of equations:

2 $$\begin{array}{ccc}\begin{array}{c}\begin{array}{c}{s}_{i}\left({d}_{i}\right)={f}_{i}\\ {s}_{i}\left({d}_{i+1}\right)={s}_{i+1}\left({d}_{i+1}\right)\end{array}\\ \begin{array}{c}{s}_{i}^{\mathrm{^{\prime}}}\left({d}_{i+1}\right)={s}_{i+1}^{\mathrm{^{\prime}}}\left({d}_{i+1}\right)\\ {s}_{i}^{\mathrm{^{\prime}}\mathrm{^{\prime}}}\left({d}_{i+1}\right)={s}_{i+1}^{\mathrm{^{\prime}}\mathrm{^{\prime}}}\left({d}_{i+1}\right)\end{array}\end{array} & \begin{array}{c}\begin{array}{c}i=0,\ldots ,n-1\\ i=0,\ldots ,n-1\end{array} \\ \begin{array}{c}i=0,\ldots ,n-1\\ i=0,\ldots ,n-1\end{array} \end{array} & \begin{array}{c}\begin{array}{c}{s}_{n-1}\left({d}_{n}\right)={f}_{n}\\ { }_{ }\end{array}\\ \begin{array}{c}{ }_{ }\\ { }_{{ }_{{ }_{{ }_{ }}}}\end{array}\end{array}\end{array},$$

For the Cubic Hermite Splines the coefficients were determined by setting the values of the Spline, and its first derivative, equal to the desired values *f*_*i*_ and $f_I^\prime$ in each node, as well as the continuity of its first derivative at the nodes in order to achieve a steady connection between the spline segments (piecewise-spline curve is C^2^ in knot). The aforementioned conditions lead to the following system of equations:

3 $$\begin{array}{ccc}\begin{array}{c}\begin{array}{c}{s}_{i}\left({d}_{i}\right)={f}_{i}\\ {s}_{i}\left({d}_{i+1}\right)={s}_{i+1}\left({d}_{i+1}\right)\end{array}\\ \begin{array}{c}{s}_{i}^{\mathrm{^{\prime}}}\left({d}_{i+1}\right)={f}_{i}^{\mathrm{^{\prime}}}\\ {s}_{i}^{\mathrm{^{\prime}}\mathrm{^{\prime}}}\left({d}_{i+1}\right)={s}_{i+1}^{\mathrm{^{\prime}}\mathrm{^{\prime}}}\left({d}_{i+1}\right)\end{array}\end{array} & \begin{array}{c}\begin{array}{c}i=0,\ldots ,n-1\\ i=0,\ldots ,n-1\end{array} \\ \begin{array}{c}i=0,\ldots ,n-1\\ i=0,\ldots ,n-1\end{array} \end{array} & \begin{array}{c}\begin{array}{c}{s}_{n-1}\left({d}_{n}\right)={f}_{n}\\ { }_{ }\end{array}\\ \begin{array}{c}{ }_{ }\\ { }_{{ }_{{ }_{{ }_{ }}}}\end{array}\end{array}\end{array}.$$

The conditions (2) and (3) allowed determining the expression (1) for each interval, leading to a set of equations that provided the appropriate value of the feedback force according to the reamer’s displacement and with the set error threshold. Cubic Hermite polynomials are more complex than the Cubic Splines, but they have no overshoots, if the data to be interpolated is not smooth as in the given case.

## Results

### Forces and torques

**Figure 3:**
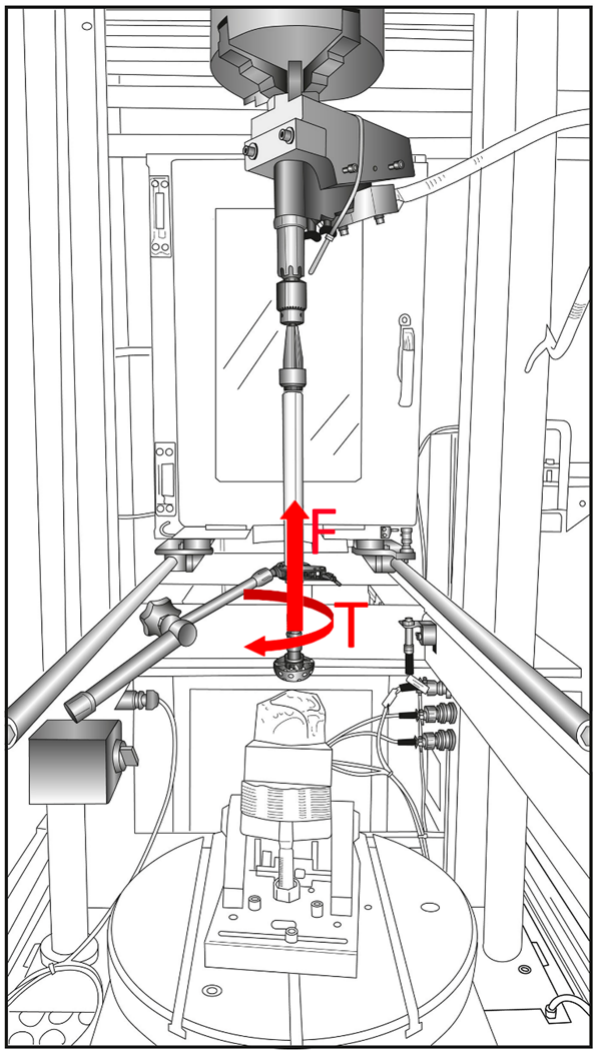
Force (F) and Torque (T) applied to the reaming tool during the tests.

Of the 72 data sets from 24 human acetabula used for the experiments, 62 resulted in valid data. Ten data sets were excluded from further evaluation due to (1) invalid measurements caused by alterations in the feed rates during the experiments (2 data sets), (2) acetabula breaking out of their resin embedding during the reaming (4 data sets) and (3) acetabula which were reamed completely after a 10-mm displacement (4 data sets). Detailed information concerning the tests results is given in the supplement material. Figure 3 shows the force and torques applied to the reaming tool during the execution of the tests.

**Figure 4:**
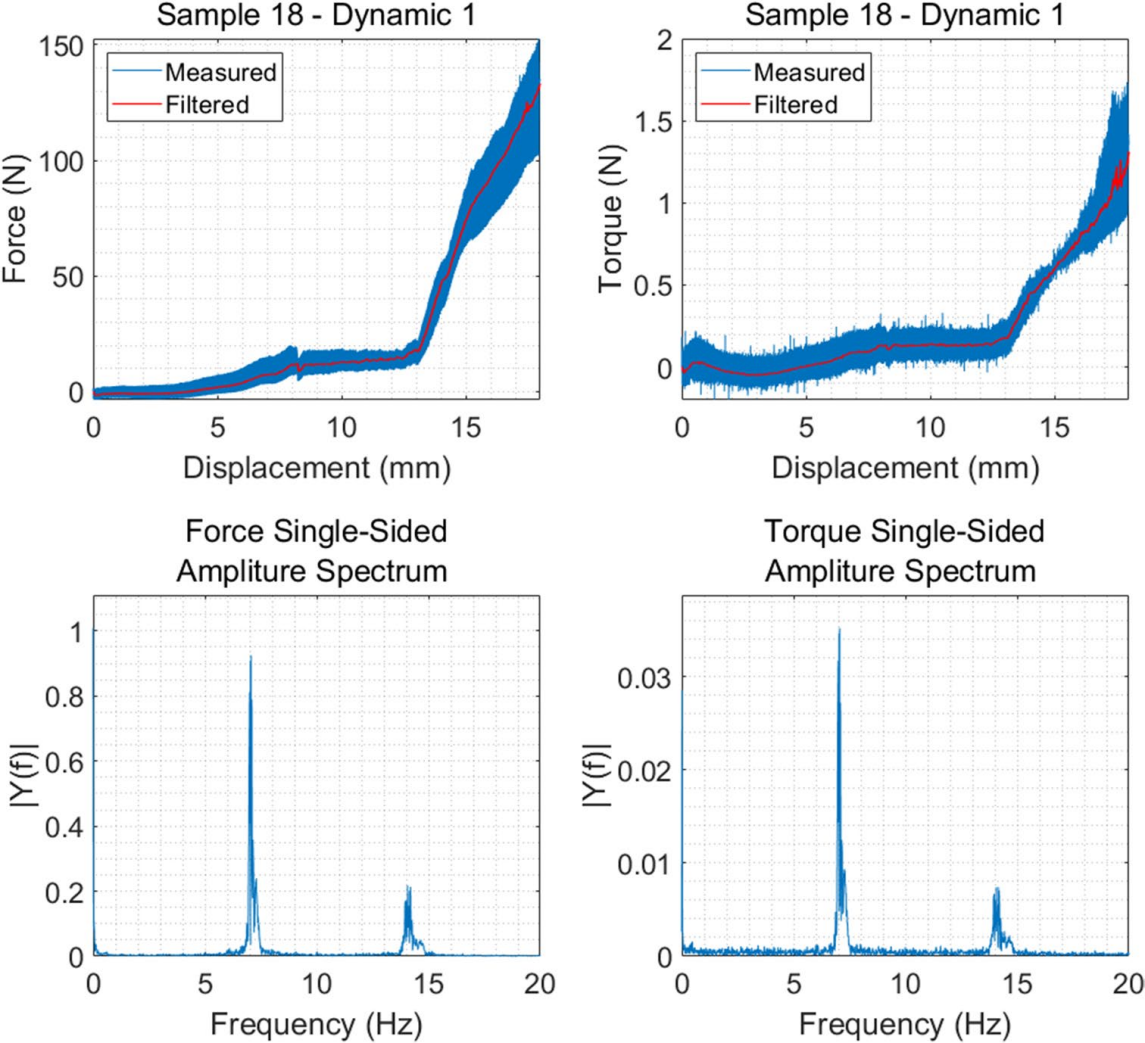
Raw and filtered force and torque measurements as well as the frequency spectrum displacement for sample 18, set Dynamic 1, feed rate 0.01 mm/s.

**Figure 5:**
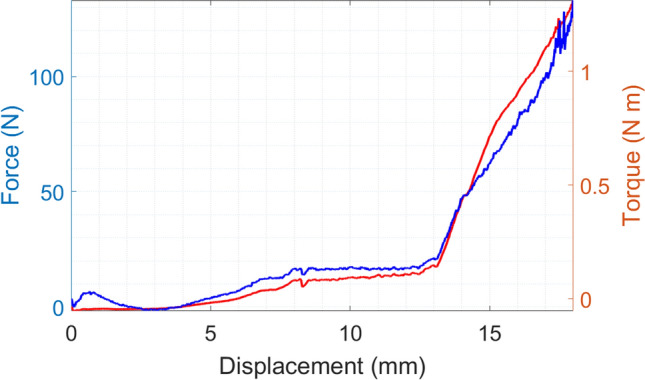
Force and torque after the filtering and the normalization with respect to the vertical displacement for sample 18, set Dynamic 1, feed rate 0.01 mm/s.

Figure 4 shows one example of force and torque measurements, before and after the filtering, alongside the frequency spectrum. Figure 5 shows an example of the normalized force and torque measurements. Similar figures for all samples are given in the supplement material.

As shown in Fig. 5, it appears that the trends of forces and torques look similar, reflecting their physical connection being consistent with the definition of torque as a product of a force for distance. The torque on the vertical axis was generated from the friction on the surface of the reamer, which is depended on the vertical force. The reason for the forces and torques remaining close to 0 during the first part of the reaming is likely caused by the slow feed rate giving the reamer sufficient time to completely remove the material so that almost no force and torque is created between the reamer and acetabulum. Further, most of the forces starting from 0 N despite the contact force of 20 N results from the minimal time difference between the start of the reamer and the start of the test program. Thus, material was removed leading to a reduction of the contact force to 0 N at the beginning of the test.

**Figure 6:**
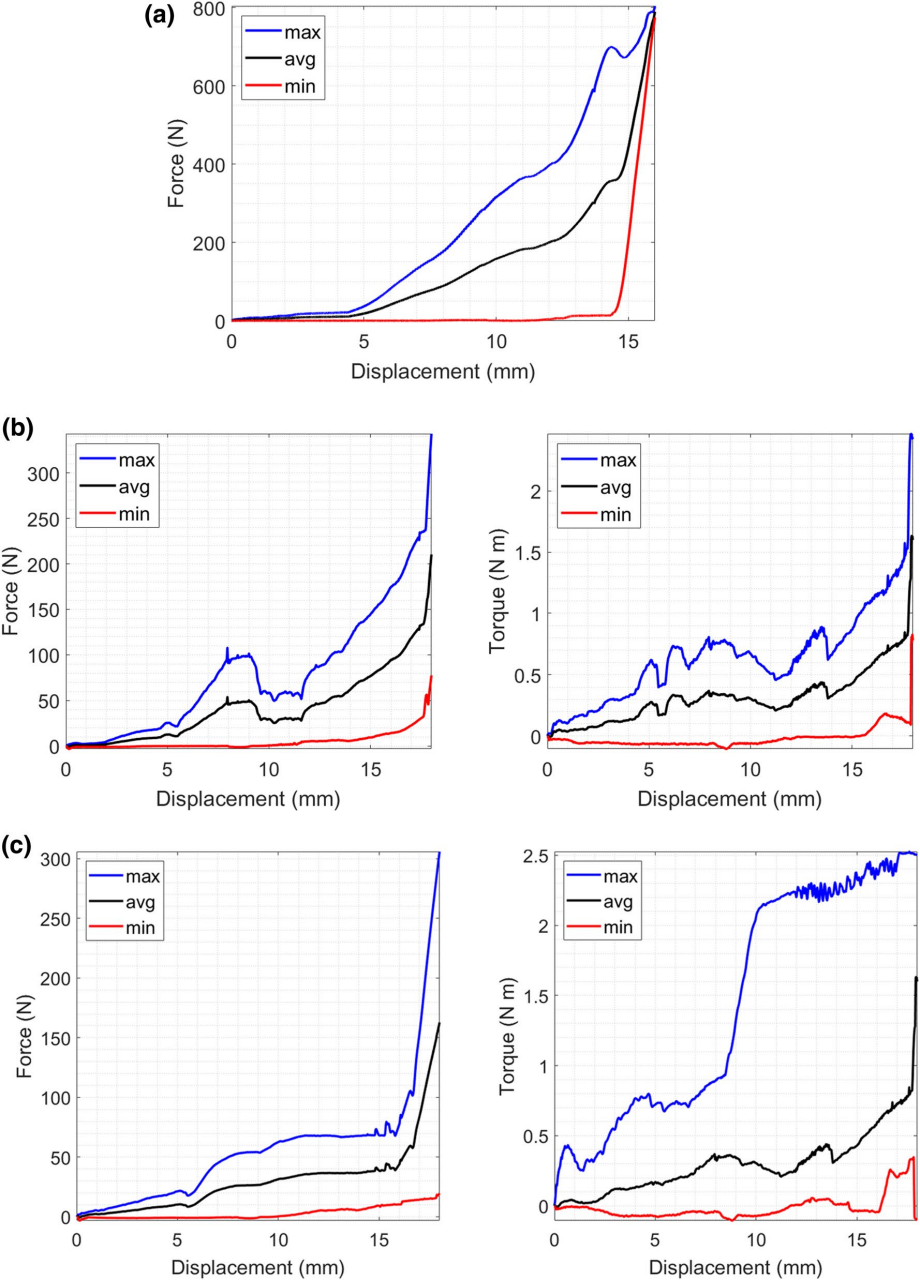
Maximum, minimum and average forces and torques for the test set Static (**a**), Dynamic 1 (**b**) and Dynamic 2 (**c**).

The maximum, average and minimum forces and torques for the test sets Static, Dynamic 1, and Dynamic 2 were calculated to provide a valid spectrum of occurring forces and torques (see Fig. 6). The depicted curves are based on the filtered and normalized data. The maximum, average and minimum curves are composed from different samples, as for each displacement value the maximum, average and minimum values at this point out of all samples were taken.

### Real-time computation of the haptic feedback using spline interpolation

In order for the VR THA simulator to deliver real-time force feedback, it is necessary that both forces and torques value are updated with a frequency of 1 kHz^15^. Therefore, on basis of the filtered and normalized data, an analytical formulation of the reamer-acetabulum interactional forces/torques, as a function of the reamer displacement, based on interpolation of the data was determined, using the algorithm described in “Data filtering, normalization and interpolation” section.

**Figure 7:**
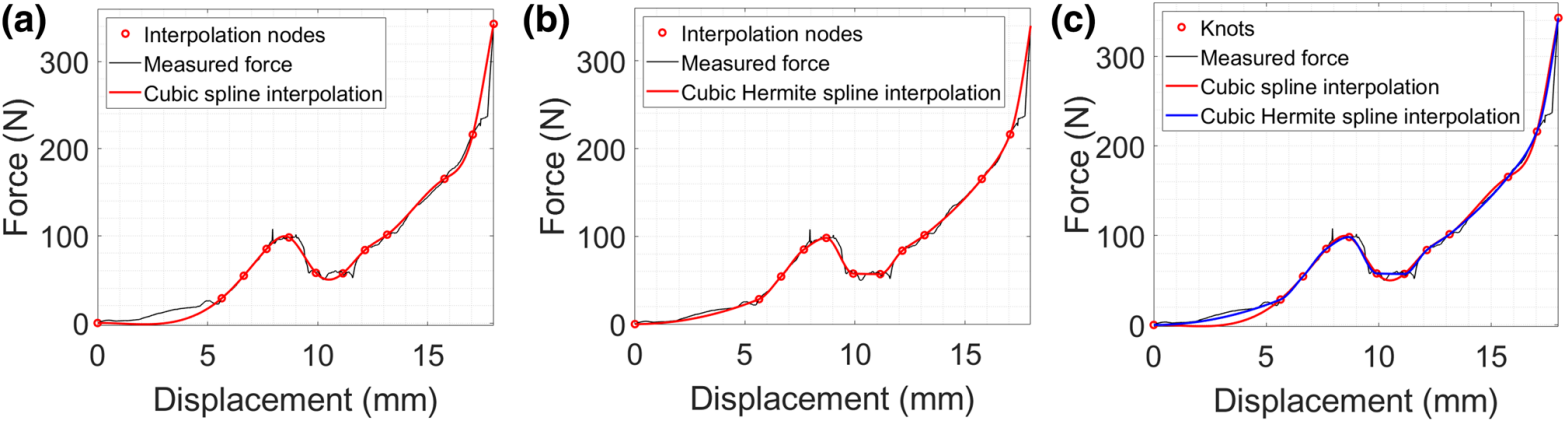
Piecewise Cubic interpolation of the maximum force feedback for the test set Dynamic 1 based on Cubic Spline (**a**) and Cubic Hermite Spline (**b**), and comparison between the interpolation techniques (**c**) in the case of 12 interpolation nodes.

Figure 7a shows an example of interpolation of the maximum force feedback for the test set Dynamic 1, based on Cubic Splines. Using the algorithm described in “Data filtering, normalization and interpolation” section, 12 interpolation nodes have been determined to achieve a maximum norm of the approximation error of 41%, corresponding at a reamer displacement of 4.7 mm. A more detailed interpolation (higher number of interpolation nodes) can be achieved reducing the norm of the approximation error.

The main advantage of Cubic Spline interpolation approach is that it produced smooth and accurate results if the data set of interpolation nodes had a smooth distribution, otherwise it presented overshooting. Cubic Hermite Spline has been determined for the same 12 interpolation nodes, in order to make a comparison between the two techniques. The smooth and accurate interpolation of the Cubic Hermite Splines resulted in a maximum norm of the approximation error of 14%, at the same reamer displacement, which was much smaller than the 41% obtained with Cubic Splines. Figure 7b shows the interpolation of the maximum force feedback for the test set Dynamic 1, based on Cubic Hermite Splines with 12 nodes.

The interpolation technique based on Cubic Hermite Splines provided a better approximation of the data for the same number of interpolation nodes. Figure 7c shows a comparison between the two interpolation techniques.

### Implementation approach

**Figure 8:**
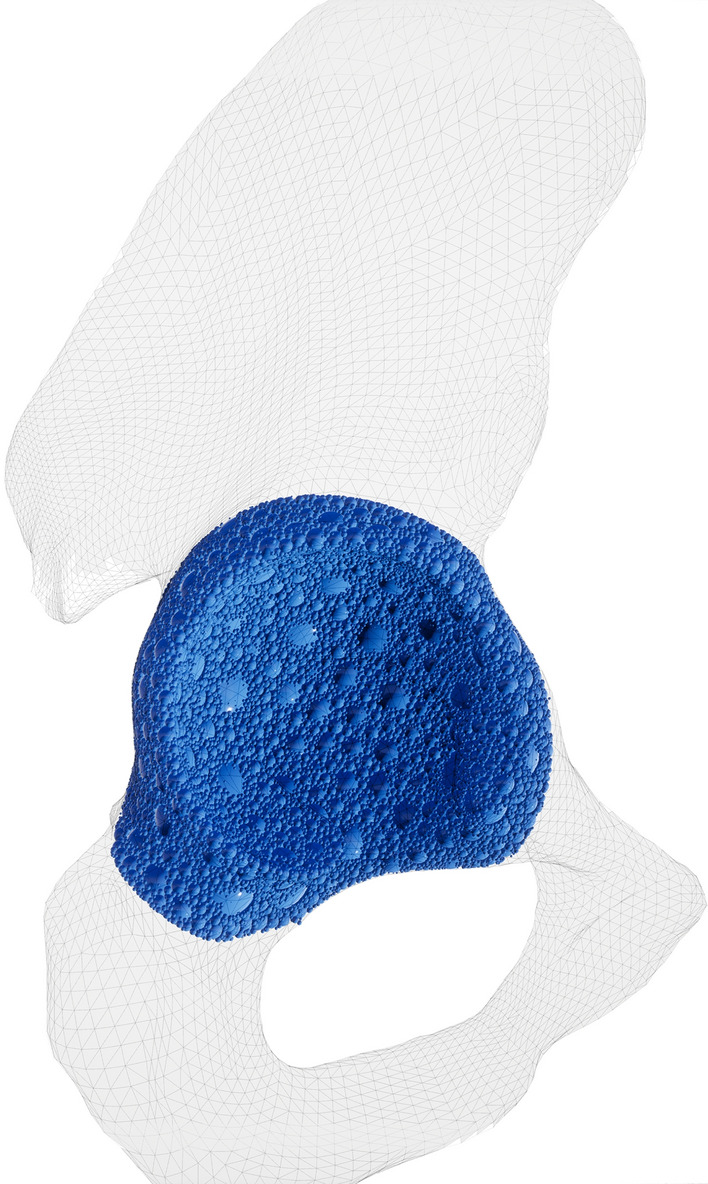
Example of a volumetric model of the acetabulum filled with spheres, which is used to calculate the force feedback given by the Virtual Reality simulator. The usage of spheres allows a fast computation of the force feedback.

To achieve a simulation model that represents the experimental data in 3D space, the hip bone mesh was converted into a volumetric representation, called sphere packing^21^. This involved filling the entire mesh with non-overlapping spheres (see Fig. 8), to optimize volume coverage. Starting from a uniform density distribution, a haptic rendering algorithm was designed, tracking a physically constraint hip reamer tool. The forces that act upon the virtual tool were rendered to the KUKA iiwa robot as force feedback. This simulation took into consideration all spheres with which the virtual reamer was in contact with and calculated a current density by weighting each sphere’s density by the contact surface between the virtual reamer and the individual spheres. The surface contact was also used to compute other surface material properties, such as friction and surface normal. The surface normal can only be reasonably approximated when all spheres within a sphere-packing are non-overlapping. Otherwise, the same geometrical features would be considered multiple times. This would incorrectly skew the resulting normal of that feature.

Based on this simulation model, a virtual experiment was established to mimic the real experiment. A virtual reamer approached a hip bone, which was fixed in space in the same angle as the physical experiment. The simulation resulted in a force curve, which gave a mean error compared to the experimental data, represented by the Cubic Hermite Spline approximation. In this way, one could relate the reamer displacement to the measured force magnitude represented by the Cubic Hermite Spline. During simulation, the density distribution of the spheres was optimized by particle swarm optimization with the goal of minimizing the error between simulated and measured force at each time step. This way an accurate volumetric model of the hip from the measured experimental data is created. The size of the spheres determines the resolution at which this optimization can operate, with smaller spheres resulting in a higher resolution. However, an increased number of spheres negatively affects the performance, therefore both effects have to be considered when choosing the sphere count. In the technical setup the material simulation was used in, the 1 kHz requirement could be achieved with 201,532 spheres^22,23^.

Additionally, the material simulation already supports material removal by splitting up, shrinking and displacing spheres. The density of each sphere is incorporated into the removal behavior, as the contact density is already calculated at runtime for the previously mentioned force feedback. Ultimately, by using the software library developed by Knopp et al.^24^, the simulation model was utilized for giving force feedback to the user with a KUKA iiwa robot.

## Discussion

The here proposed approach represents a first attempt to develop a cadaver-based real-time material model which can be implemented into a haptic VR-based THA surgery simulator. For this purpose, an industrial KUKA iiwa robotic arm (KUKA AG, Augsburg, Germany) was deployed as a force feedback device. The aim was to deliver a realistic haptic feeling when virtually reaming the acetabulum. During real THA, the forces applied by the surgeon for the reaming of the hip exceed what can be delivered by standard commercial type haptic devices available on the market at present. We here made attempt to resolve all these issues for a more realistic VR reaming simulation. Another challenge in the development of surgical VR simulators lies in the delivery of realistic force feedback, as it would be felt when manipulating, cutting or milling human tissues. To achieve these goals, it was necessary to measure the biomechanical behavior of the acetabulum when reamed in human cadavers. Industrial robotic arms are capable of delivering much higher forces, as is the case in THA or similar musculoskeletal surgery. The approach comes with alienating the industrial robot not to just perform standard handling operations but to actively provide a counter force for the force applied by the surgeon via a reaming tool upon it, simulating what is experienced during THA surgery. A simulation model, which implemented the approach described in “Implementation approach” section is depicted in Fig. 8. Here, spheres have been integrated into the acetabular region to effectively minimize computational time, given that intersections between spheres are the fastest computational method.

Different from other fields in engineering, the test procedures and the underlying test setup for determining the material properties of the reaming were neither defined nor standardized. Therefore, we developed a new standardized testing procedure to assess the biomechanical response of the acetabula when reamed with a real surgical instrument. Instead of multiple angles as it would be the case during surgery, we only used one direction for standardization purposes. This resulted in highly accurate and consistent data of the interactional forces and torques between the surgical instrument and the acetabula. Further, our here presented standardized test procedure could also be applied to other drilling or reaming scenarios where interactional forces and torques between a surgical instrument and tissue need to be measured, e.g. the drilling of bone channels for inserting bone screws. The specific nature of the load and torque data being non-uniform and involving one characteristic local peak at this stage is not well understood and is lacking the morphological link to the here presented mechanical data. Potentially, the diameters of the acetabulum and reaming device, inhomogeneities in cartilage thickness as well as the hip not being a perfect sphere may be causes for the here observed phenomena. This explanation, however, remains unsubstantiated and warrants further study in future projects.

A common approach for developing a material model simulating interactional forces and torques between the reamer and the acetabulum would use FEA. However, to develop such an FEA model, measurements with far more than the here available 24 human acetabula would be necessary to determine the influence of parameters like age, sex, bone density and thicknesses of the different tissue layers. Additionally, in mechanical engineering, tests aimed at determining material properties to be implemented in FEA are starting from a known and standardized testing procedure and sample geometry. In the given case, the testing procedure had to be developed first and the geometry of each acetabulum is well known to be different inter-individually. However, even if an FEA model simulating the interactional forces and torques between the reamer and the acetabulum existed, it could not be used in a surgical VR training simulator: Haptic feedback needs an update rate of 1 ms, which is usually out of reach of any contemporary FEA simulation, because of the computational load.

For this reason, the here given approach has been to measure directly the interactional forces and torques between the reamer and the acetabulum and deriving an analytical representation as a function of the reamer displacement. However simply using polynomials for interpolating the measured curves would have led to polynomials of high orders consuming considerable computational power. Therefore, the here presented approach uses Cubic Splines to interpolate piecewise the measured forces/torques with an appropriate number of interpolation nodes. Further, piecewise interpolation based on Cubic Splines avoids problems associated with simpler solutions like the Lagrange approach because they avoid Runge’s phenomenon.

However, by using Cubic Splines, one can run into computational overload depending on the number of nodes needed for the interpolation. Here an optimization conflict arises between the fitting of the interpolated curve to the measured data (many nodes) and the computational resources it requires (few nodes). This work presented an approach to determine the appropriate number of nodes by analyzing the second derivative of the measured quantities together with the maximum tolerable error between the measured data and the interpolated curves.

Additionally, we compared Cubic Hermite Splines with Cubic Splines to increase the fitting quality with lesser node numbers. The results showed that Cubic Hermite Splines in general provide lower error between the interpolated curve and the measured data than Cubic Splines using the same number of nodes. By using Cubic Hermite Splines, one could further avoid the problem of the deviation of the interpolated curve from the data set as the number of nodes decreases, leading to more accurate results. Even with a decreasing number of interpolation nodes, a better approximation is achieved using Cubic Hermite Splines. This indicates, in order to achieve a defined approximation using the Cubic Hermite Splines, it is possible to use a reduced number of interpolation nodes, requiring lower computational power.

**Figure 9:**
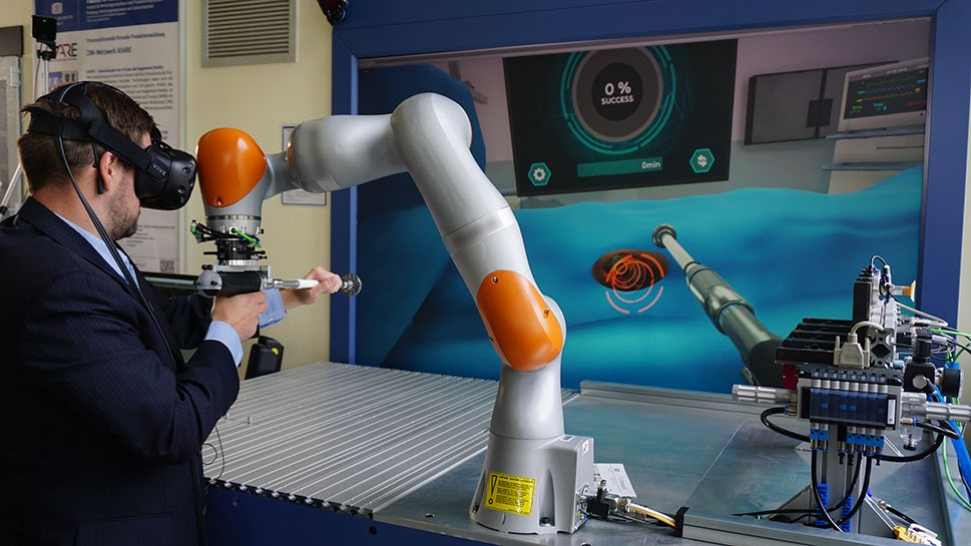
The Virtual Reality acetabulum reaming simulator, where the developed real-time material model is used to simulate the interactional forces and torques between the reamer and the acetabulum.

It could further be shown that using the given approach, a valid spectrum of occurring forces and torques can be obtained, which can be used for the simulation of forces and torques during reaming in VR simulation. As the here developed material model bases on Cubic Hermite Splines, the level of machining hardness of the virtually-reamed acetabulum can be freely defined within a given range. These combined efforts will allow the training surgeons to experience different acetabula in the VR acetabulum reaming simulator developed by our group^25,26^ (see Fig. 9). However, conclusions on the realism of the haptic behavior cannot be drawn and need to be evaluated in conjunction with the entire VR training experience in a user study with orthopedic surgeons. Such future user study should target advanced students in medical schools or allied disciplines, residents and experienced surgeons, to get a holistic overview of the expectations of all relevant stakeholders. Medical students may provide their anticipated expectations on the training simulator. Residents may express their needs based on their currents training experiences with state-of-the-art training methods. Experienced surgeon may best assess the quality of the training simulator and can evaluate the training concept. The user study should measure user experience, usability, and the cognitive work load of the simulator using established post-test questionnaires. Further presence should be measured with short post-test questionnaire as well. As a last quantitative measure, a self-developed post-test questionnaires about the quality and the expected capacity of the simulator for each participant groups should be used. This to be developed questionnaire should base on and adapt questionnaire from literature used to asses other VR surgery trainings simulator, e.g. for minimally-invasive surgeries. An interview with each participant about their training experience should conclude the assessment.

Further, the here obtained data sets could be used as a part of a training data set to develop a deep neural network (DNN) based material model, which was not the aim of the study but presents interesting future work. However, generating a good DNN based material model would require much more data sets then we could acquire. Given that such a DNN based material model would exist, it would be interesting to compare the felt realism of the generated haptic force feedback in a user study.

### Limitations

A number of limitations exist related to the given work: First, the number of acetabula available for the tests was limited to 24 samples, with a narrow geriatric age range and only one angle of reamer rotation. Though the age range of the cadavers assessed here may not exactly fit with the age range of patients undergoing THA, these first baseline data may well serve as a reference for ranges of material properties of the acetabulum when reamed. With the limited number of acetabula, it was only possible to obtain data on the general spectra of forces and torques occurring during the reaming of human acetabula. For training purposes, it would be desirable to train patient group specific reaming behavior depending on age, sex and bone quality. To achieve this, more data sets are needed. Second, the interactional forces and torques were measured along one (reaming tool) direction. Future work should repeat the given experiments, obtaining mechanical data at different reaming angles similar to the acquisition of a bidirectional reflectance distribution function for the rendering of graphics models to improve the material model. Third, the acetabula were casted in a rigid support, thus, the influence of soft tissues surrounding the acetabulum was not reflected in the measured biomechanical data. A fourth possible limitation is that the Spline interpolation leads to a smooth material behavior. A solution for this would be to reduce the maximum error leading to higher numbers of control point with a better representation of the measured jitter. Alternatively, the presented Cubic Hermite Spline material model could be extended with a noise factor introducing the measured jittering behavior to the material model. A user study determining the felt realism of the presented model is necessary to evaluate if the smoothened behavior of the presented model poses a problem.

## Conclusion

We here presented for the first time a fast computing material model of the human acetabulum, which is applicable for haptic reaming simulators, as is required for VR THA surgery training. A standardized biomechanical testing setup was established to measure the interactional forces and torques during the reaming of acetabula, as is performed during THA. Based on tests with human acetabula under static and dynamic settings, a fast computing Cubic Hermite Splines based material model was developed. It was shown that Cubic Hermite Splines provided a superior approximation of the real forces and torques compared to Cubic Splines. Finally, the implementation of a Cubic Hermite Splines material model combined with the here measured spectra of interactional forces and torques into a simulator will allow surgeons to train with a variety of machining hardness levels of acetabula of haptic VR acetabulum reaming (Supplementary Information 1).

## Supplementary information

Supplementary Information 1.
