# Supplementary material for: A cadaver-based biomechanical model of acetabulum reaming for surgical virtual reality training simulators
Source: Sci Rep. 2020 Sep 3;10:14545. doi: 10.1038/s41598-020-71499-5 (PMC7471911; doi:10.1038/s41598-020-71499-5)
Supplement: Supplementary file 1 — Supplementary Information 1. [file 41598_2020_71499_MOESM1_ESM.docx]

A cadaver-based biomechanical model of acetabulum reaming for surgical virtual reality training simulators

**Luigi Pelliccia^1,^*, Mario Lorenz ^1,2,5,^*, Christoph-E. Heyde^2^, Maximilian Kaluschke^3^, Philipp Klimant^1,4^, Sebastian Knopp^1^, Stefan Schleifenbaum^2^, Christian Rotsch^2,4^, René Weller^3^, Michael Werner^4^, Gabriel Zachmann^3^, Dirk Zajonz^2^, Niels Hammer^2,4,5^**

*** Equal contributors**

^1^ Chemnitz University of Technology

Professorship Machine Tool Design and Forming Technology

Professorship Factory Planning and Factory Operation

Reichenhainer Straße 70

09126 Chemnitz, Germany

E-mail: (mario.lorenz, luigi.pelliccia, philipp.klimant, sebastian.knopp)@mb.tu-chemnitz.de

^2^ University Hospital Leipzig

Department of Orthopedics, Trauma and Plastic Surgery

Liebigstraße 20

04103 Leipzig, Germany

E-mail: {christoph-eckhard.heyde, stefan.schleifenbaum, dirk.zajonz}@medizin.uni-leipzig.de

^3^ University of Bremen

Department of Computer Graphics and Virtual Reality

Bibliothekstraße 5

28359 Bremen, Germany

E-mail: {mxkl, weller, zach}@cs.uni-bremen.de

^4^ Fraunhofer Institute for Machine Tools and Forming Technology IWU

Nöthnitzer Straße 44

01187 Dresden, Germany

E-mail: {christian.rotsch, michael.werner}@iwu.fraunhofer.de

^5^ Medical University of Graz, Chair

Department of Macroscopic and Clinical Anatomy, Chair

Harrachgasse 21

8010 Graz, Austria

E-mail: nlshammer@googlemail.com

**Supplement materials**

**Static sample group**

*
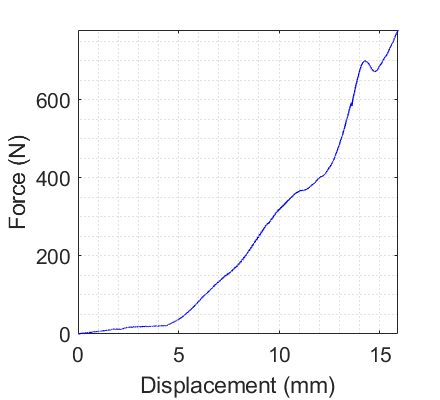
***
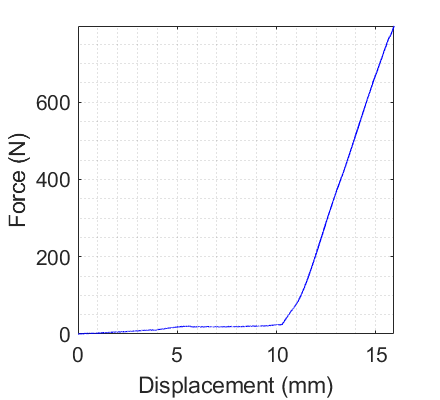
**

Figure 1. Sample 1 (left) and sample 2 (right)

**
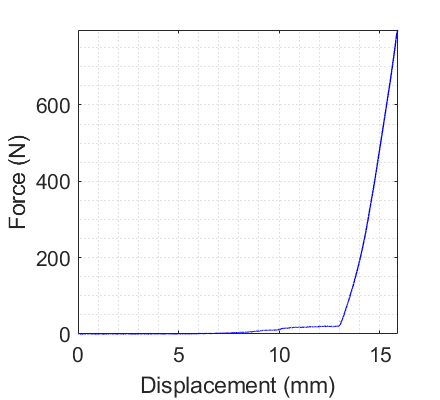

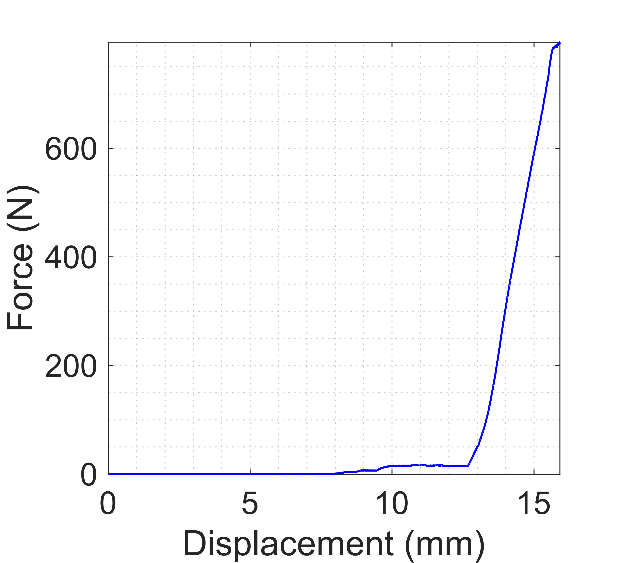
**

Figure 2. Sample 3 (left) and sample 4 (right)

**
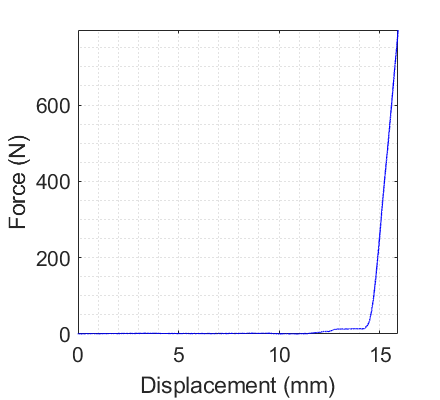

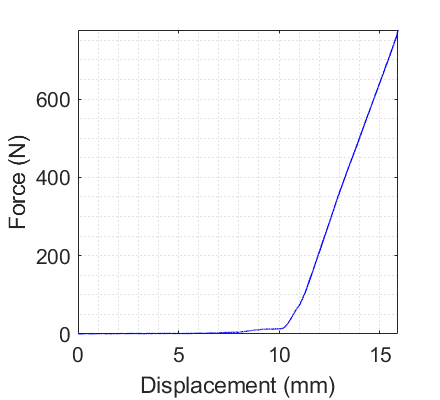
**

Figure 3. Sample 5 (left) and sample 6 (right)

**
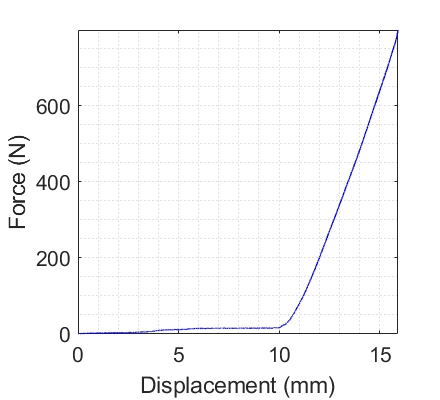

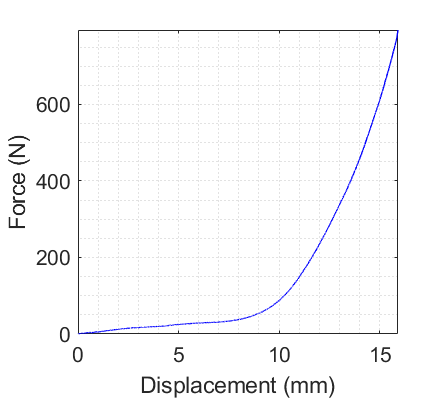
**

Figure 4. Sample 7 (left) and sample 8 (right)

**
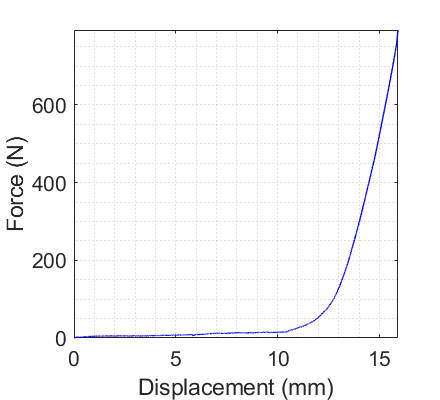

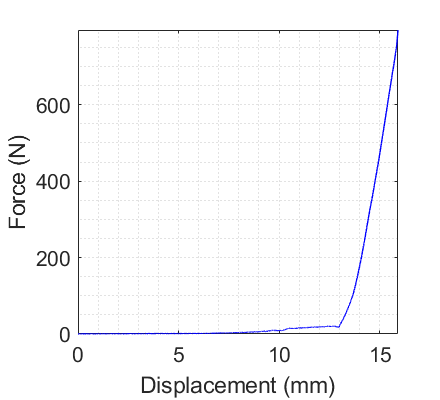
**

Figure 5. Sample 9 (left) and sample 10 (right)

**
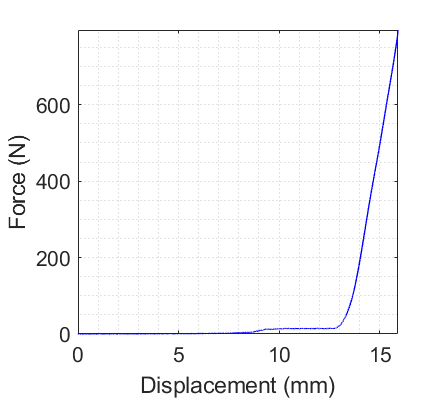

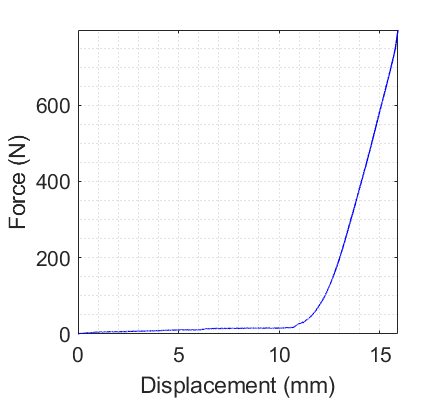
**

Figure 6. Sample 11 (left) and sample 12 (right)

**
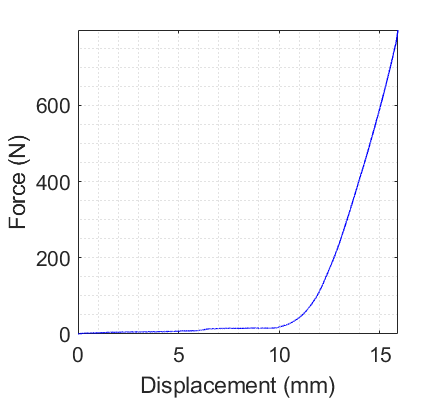

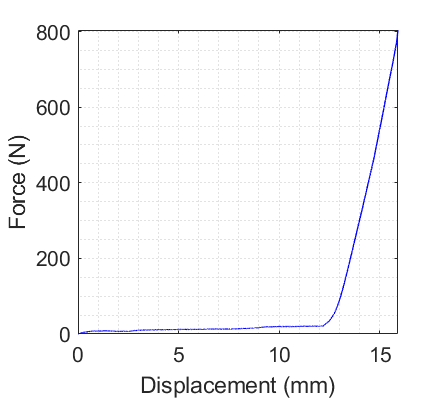
**

Figure 7. Sample 13 (left) and sample 14 (right)

**
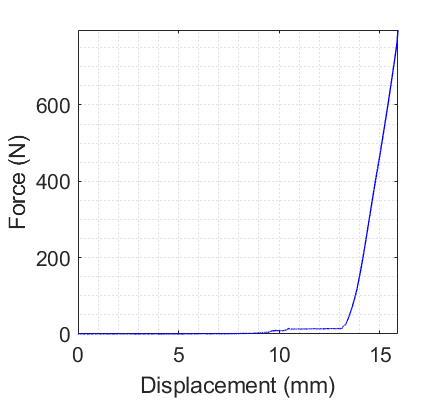

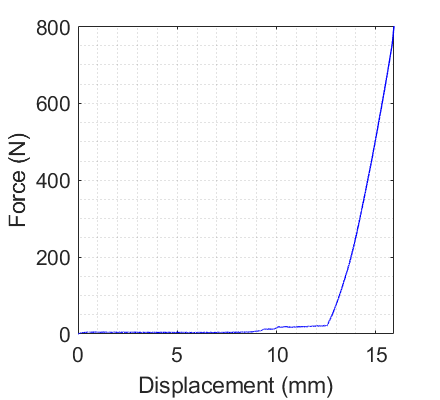
**

Figure 8. Sample 15 (left) and sample 16 (right)

**
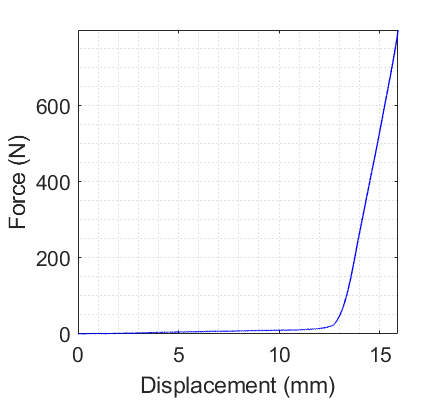

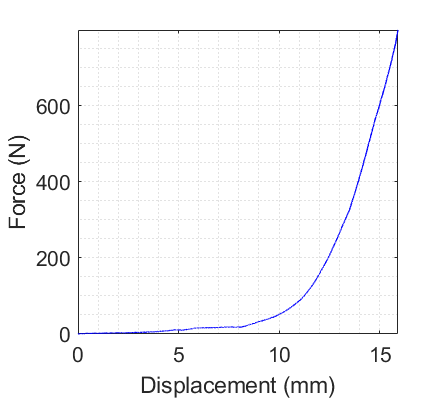
**

Figure 9. Sample 17 (left) and sample 18 (right)

**
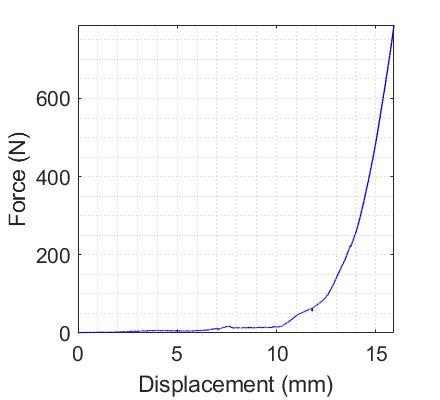

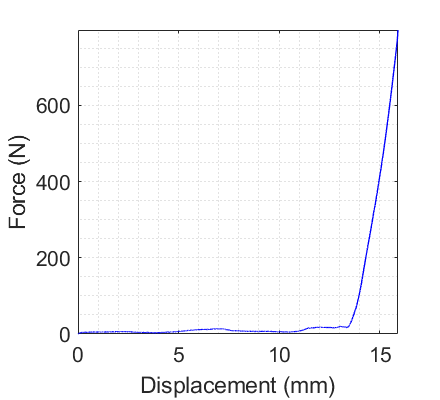
**

Figure 10. Sample 19 (left) and sample 20 (right)

**
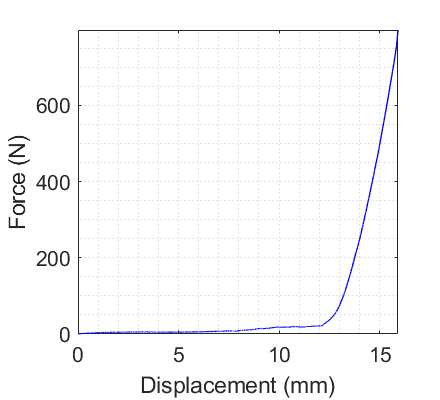

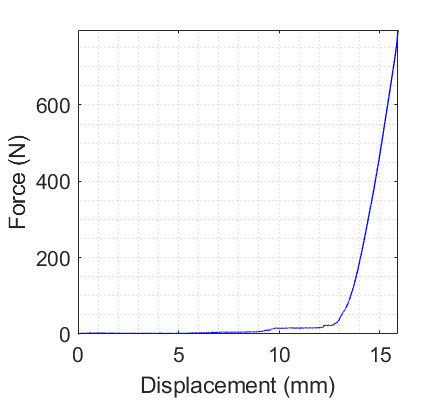
**

Figure 11. Sample 21 (left) and sample 22 (right)

**
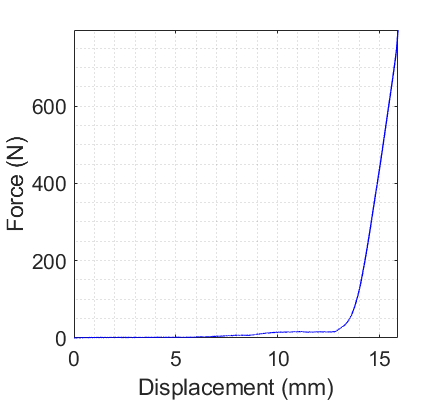

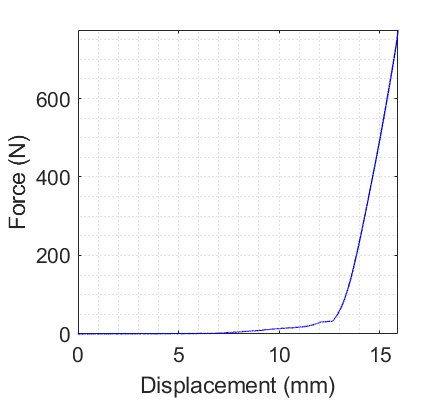
**

Figure 12. Sample 23 (left) and sample 24 (right)

Figures 1 to 12. Displacement-force curves are given for each of the acetabular bone samples trialed.

**First dynamic sample group (Dynamic 1):**

Table 1 lists the samples used for the first dynamic set. The tests are numbered according to the sequence adopted from the test protocol. Details of the excluded samples can be found in the results section.

| Sample number | Feed rate (mm/s) | Sex | Age (years) | Side | Tool diameter (mm) |
| --- | --- | --- | --- | --- | --- |
| 2 | 0.03 | Female | 102 | Left | 44 |
| 3 |  | Male | 82 | Right | 52 |
| 5 |  | Female | 91 | Right | 52 |
| 6 |  | Female | 102 | Right | 46 |
| 7 |  | Female | 78 | Right | 50 |
| 8 |  | Female | 78 | Right | 50 |
| 9 |  | Female | 87 | Left | 46 |
| 10 |  | Male | 91 | Left | 52 |
| 11 |  | Female | 93 | Left | 46 |
| 12 |  | Male | 84 | Right | 54 |
| 13 | 0.01 | Female | 89 | Left | 48 |
| 14 |  | Female | 78 | Left | 48 |
| 17 |  | Female | 91 | Left | 48 |
| 18 |  | Female | 89 | Right | 48 |
| 19 |  | Male | 85 | Right | 52 |
| 20 |  | Female | 74 | Left | 50 |
| 23 |  | Male | 82 | Left | 48 |
| 24 |  | Male | 80 | Left | 50 |

Table 1. First dynamic sample data set (Dynamic 1) for both feed rates

***Feed rate 0.01 mm/s***

***
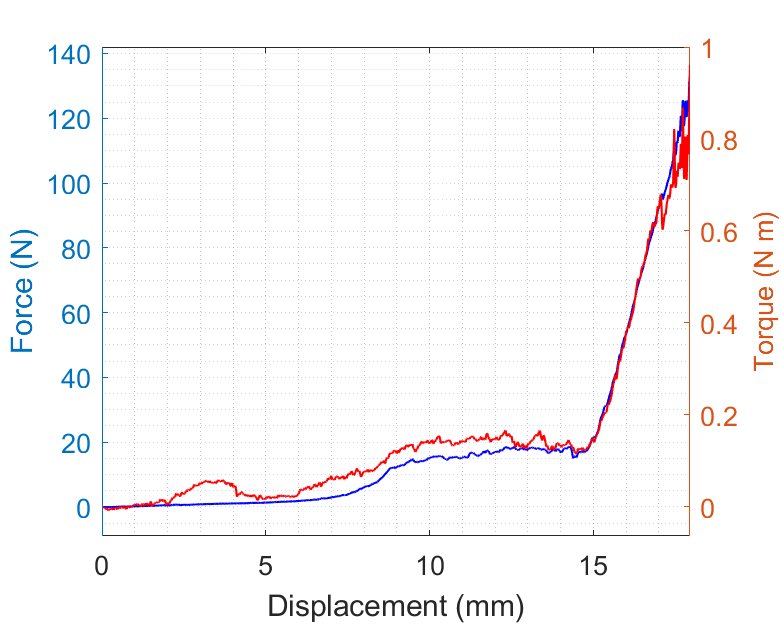
*** ***
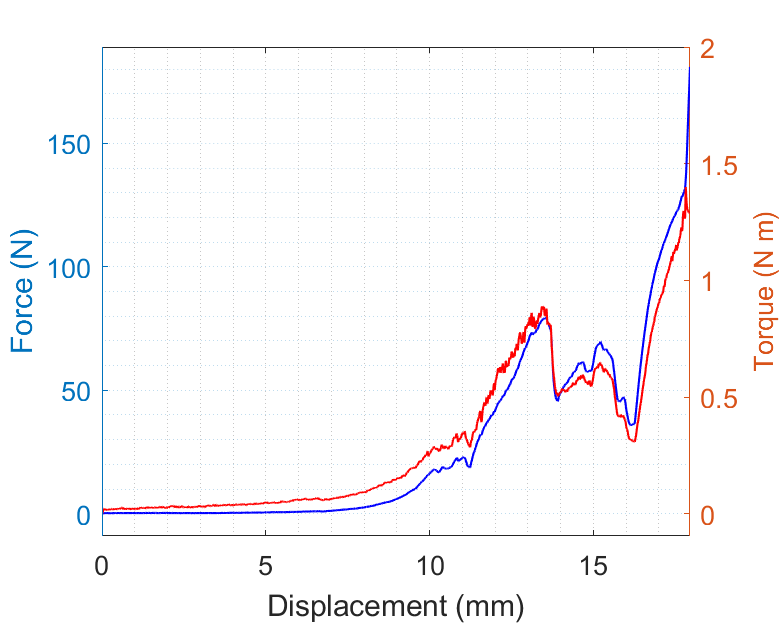
***

Figure 13. Sample 23 (left) and sample 13 (right)

***
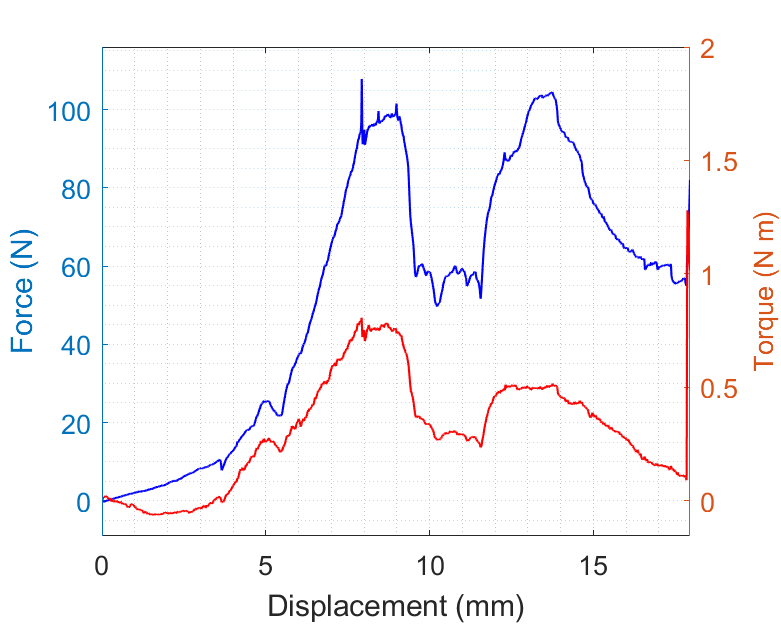
*** ***
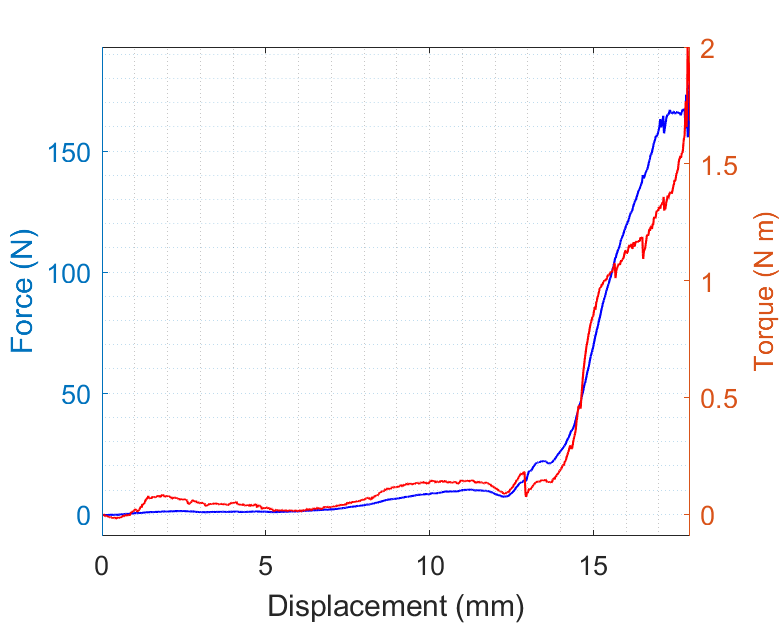
***

Figure 14. Sample 14 (left) and sample 19 (right)

***
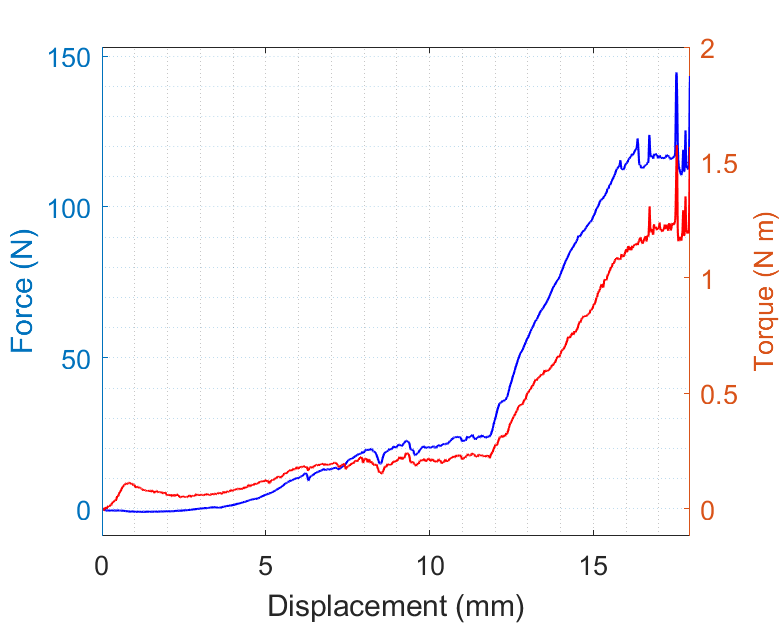
*** ***
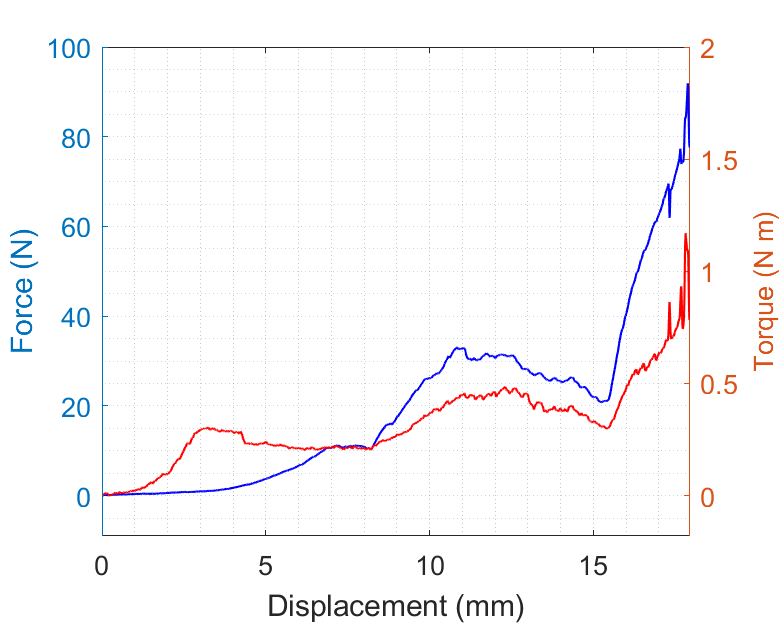
***

Figure 15 Sample 17 (left) and sample 24 (right)


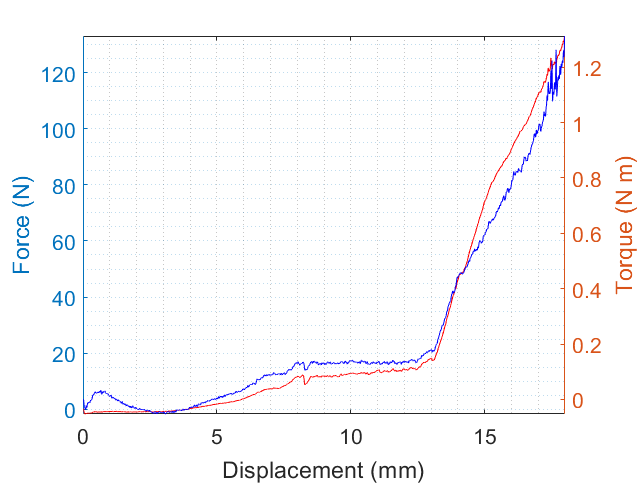

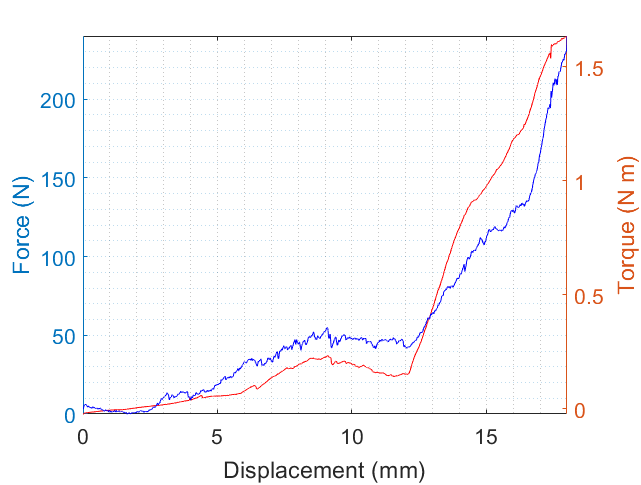


Figure 16 Sample 18 (left) and sample 20 (right)

***Feed rate 0.03 mm/s***


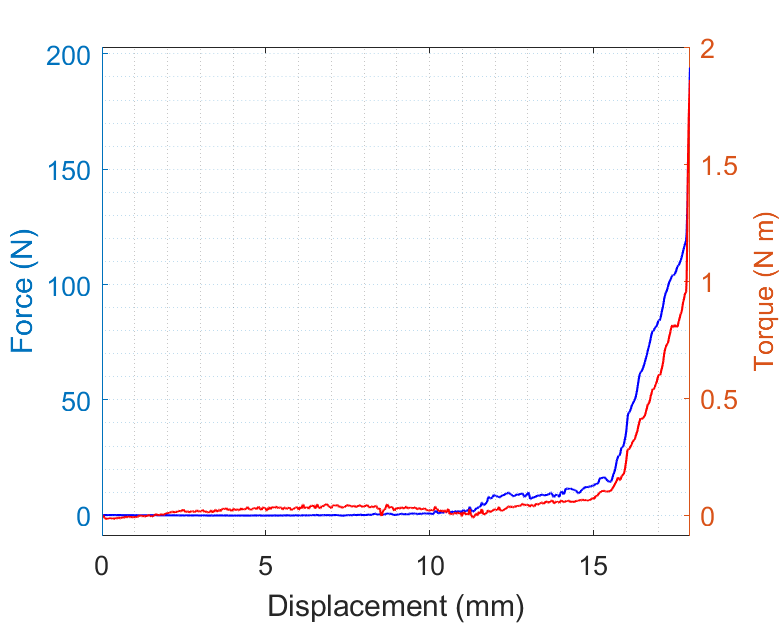
 *
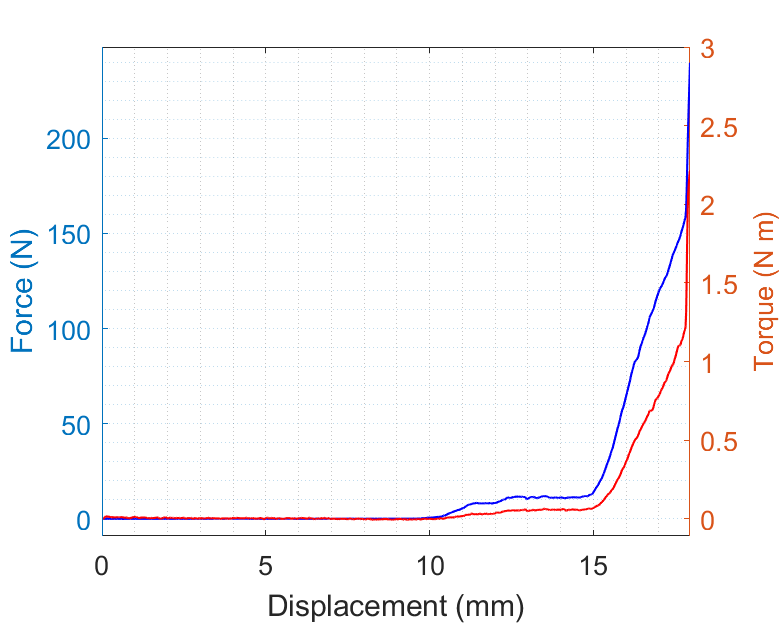
*

Figure 17. Sample 2 (left) and sample 3 (right)

*
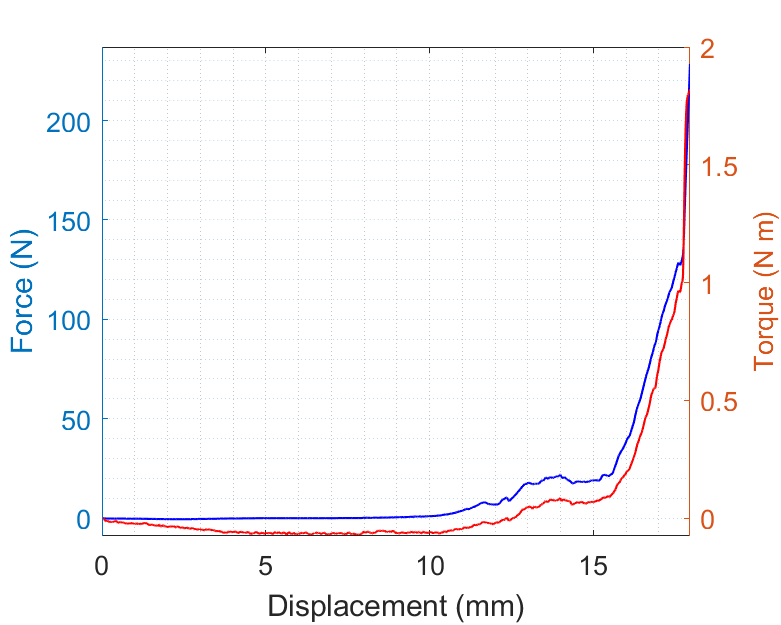
*
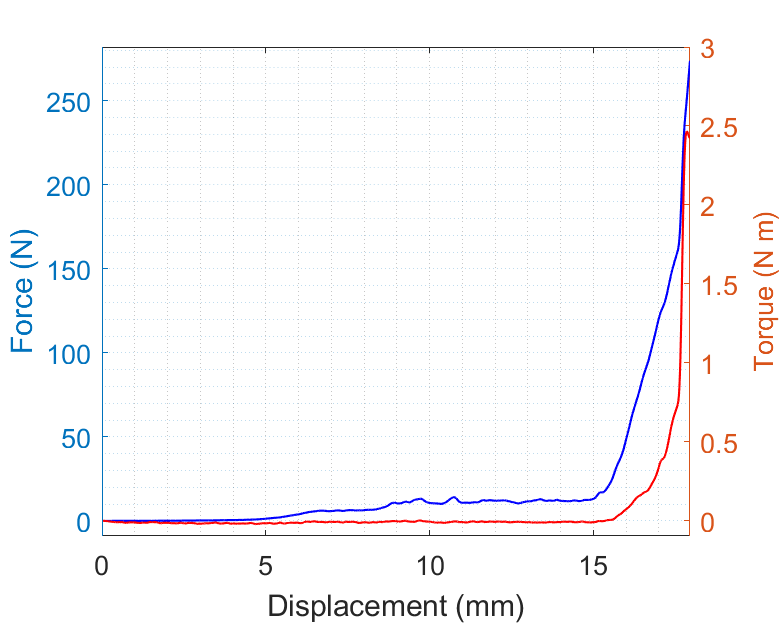


Figure 18. Sample 5 (left) and sample 6 (right)

*
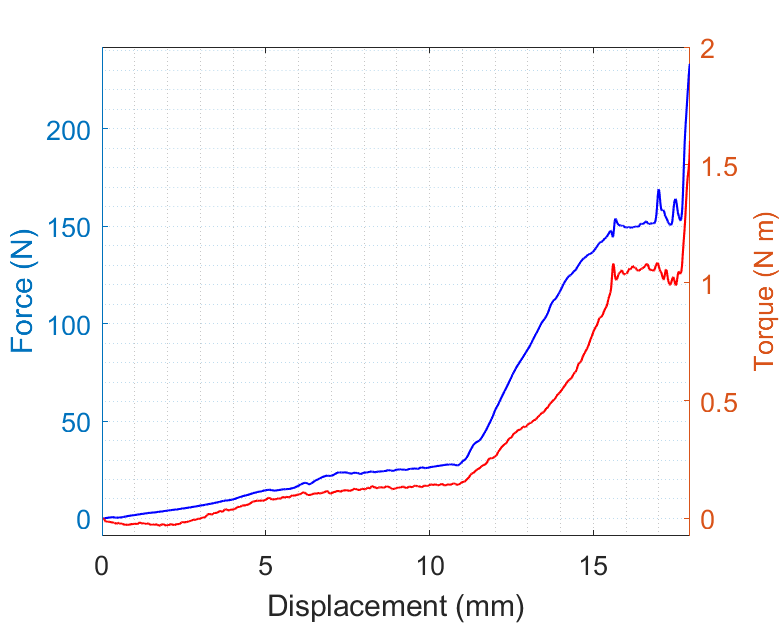
* *
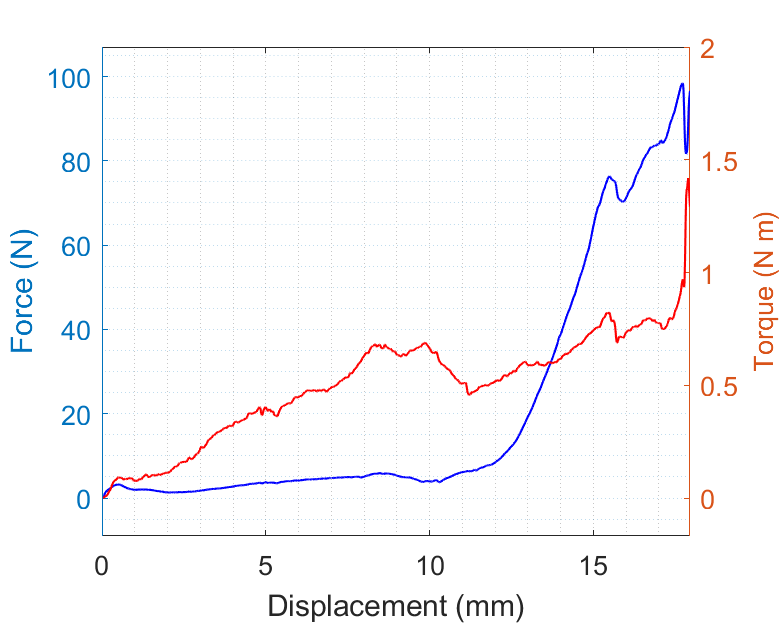
*

Figure 19. Sample 7 (left) and sample 8 (right)

*
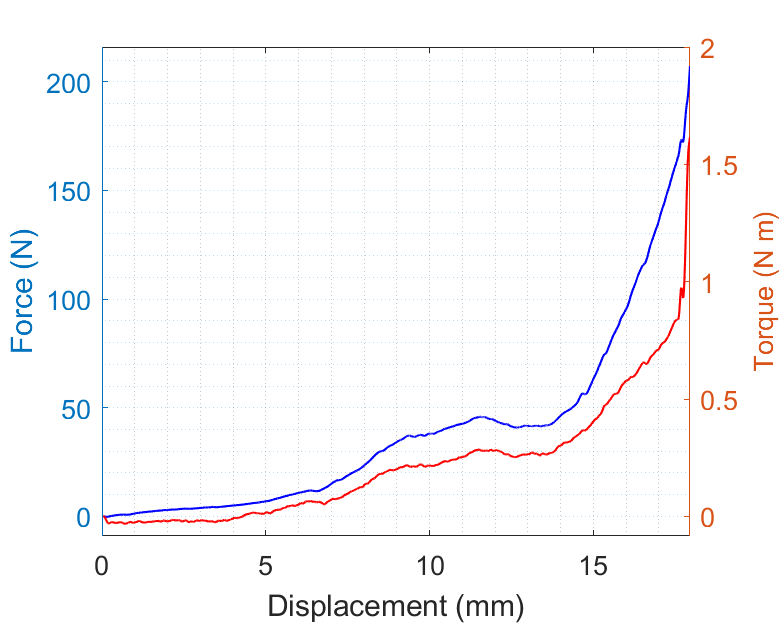
*
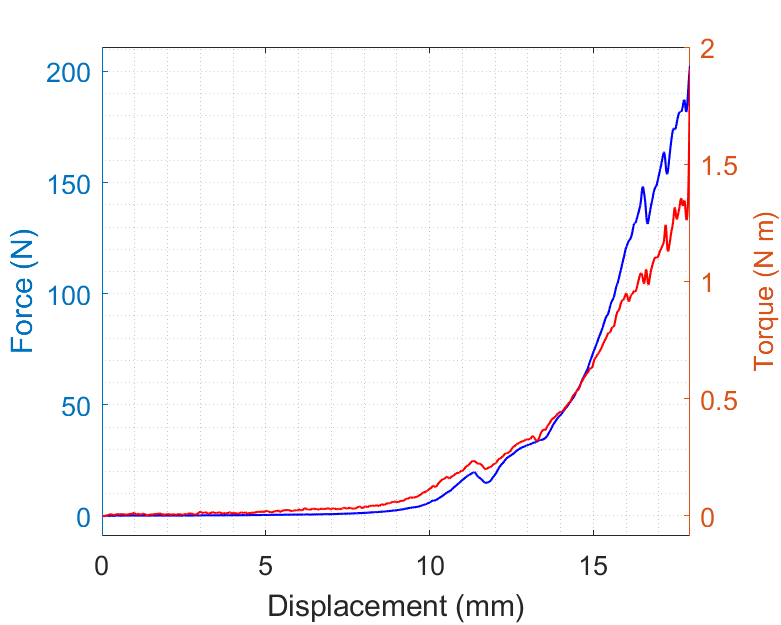


Figure 20. Sample 9 (left) and sample 10 (right)

*
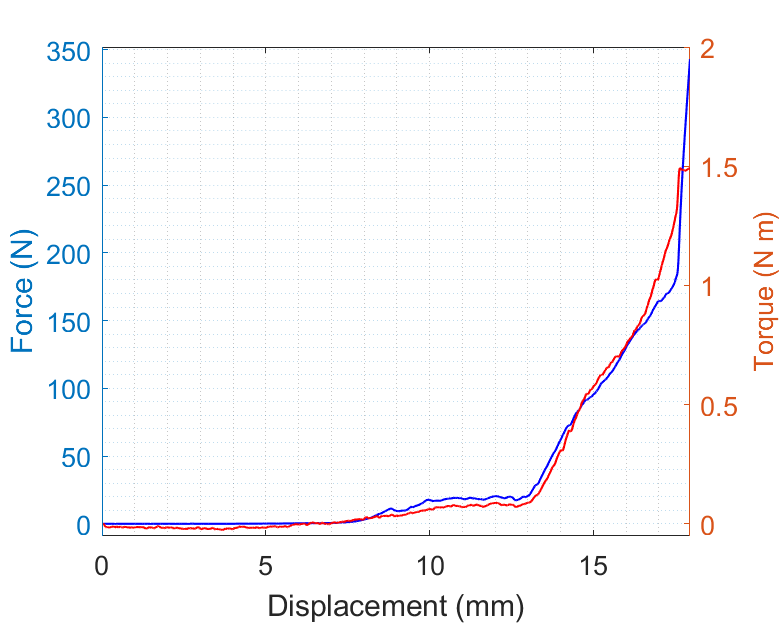
*
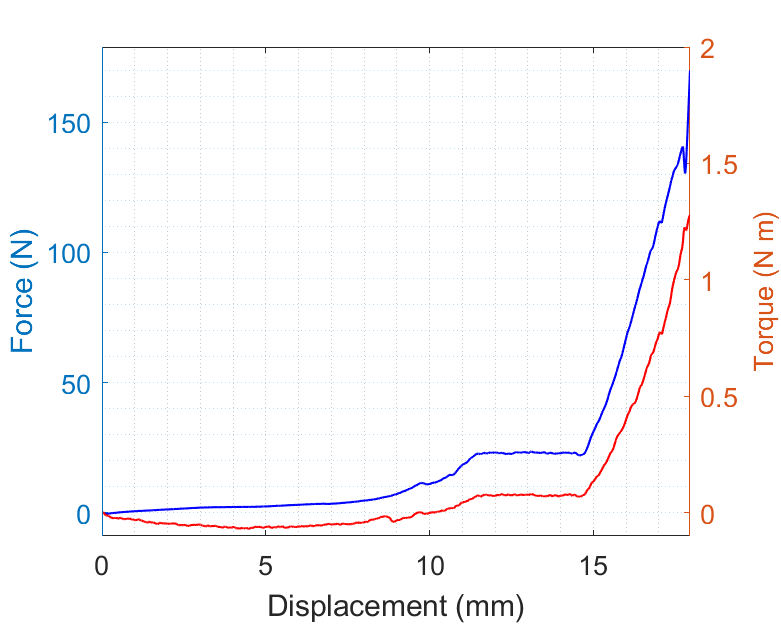


Figure 21. Sample 11 (left) sample 12 (right)

**Second dynamic sample group (Dynamic 2):**

Table 2 summarizes the samples used for the second dynamic set. The tests are numbered according to the sequence adopted from the test protocol. Details of the excluded samples can be found in the results section

| Sample number | Feed rate (mm/s) | Sex | Age (years) | Side | Tool diameter (mm) |
| --- | --- | --- | --- | --- | --- |
| 2 | 0.03 | Female | 102 | Left | 46 |
| 3 |  | Male | 82 | Right | 54 |
| 5 |  | Female | 91 | Right | 54 |
| 6 |  | Female | 102 | Right | 48 |
| 7 |  | Female | 78 | Left | 52 |
| 8 |  | Female | 78 | Right | 52 |
| 9 |  | Female | 87 | Left | 48 |
| 10 |  | Male | 91 | Left | 54 |
| 11 |  | Female | 93 | Left | 48 |
| 12 |  | Male | 84 | Right | 56 |
| 13 | 0.01 | Female | 89 | Left | 50 |
| 14 |  | Female | 78 | Left | 50 |
| 15 |  | Female | 98 | Right | 46 |
| 17 |  | Female | 91 | Left | 50 |
| 18 |  | Female | 89 | Right | 50 |
| 19 |  | Male | 85 | Right | 54 |
| 20 |  | Female | 74 | Left | 52 |
| 23 |  | Male | 82 | Left | 50 |
| 24 |  | Male | 80 | Left | 56 |

Table 2. Second dynamic sample data set (Dynamic 2) for both feed rates

***Feed rate 0.01 mm/s***


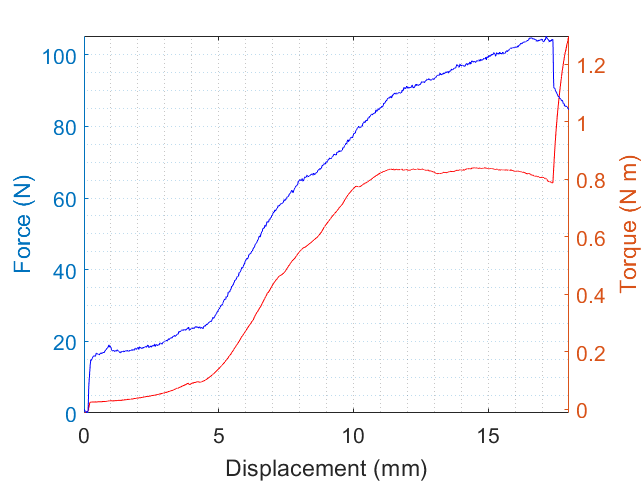

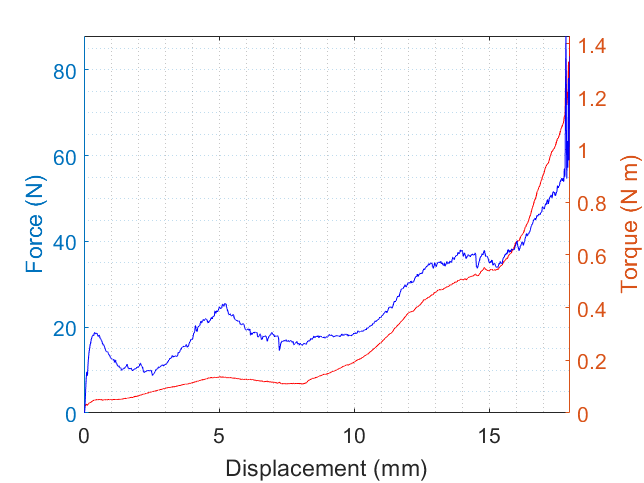


Figure 22 Sample 14 (left) sample 17 (right)


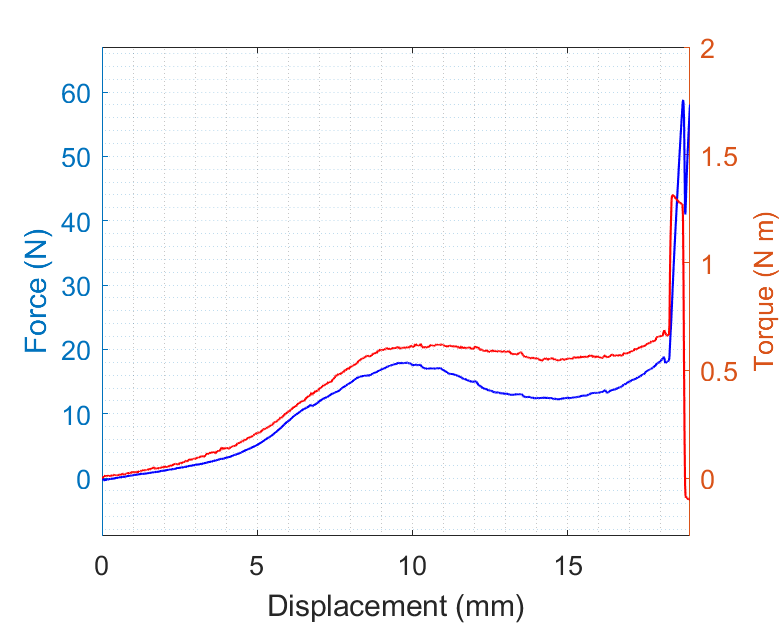

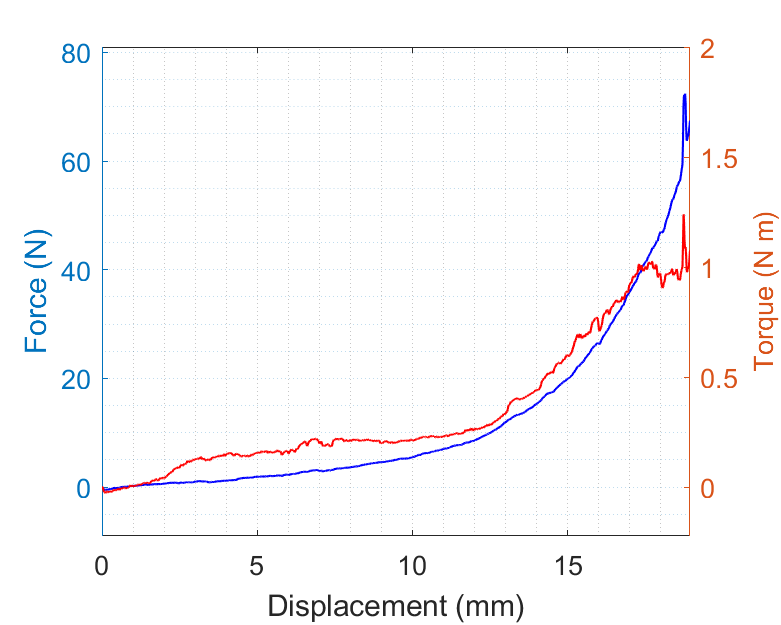


Figure 23. Sample 13 (left) sample 15 (right)


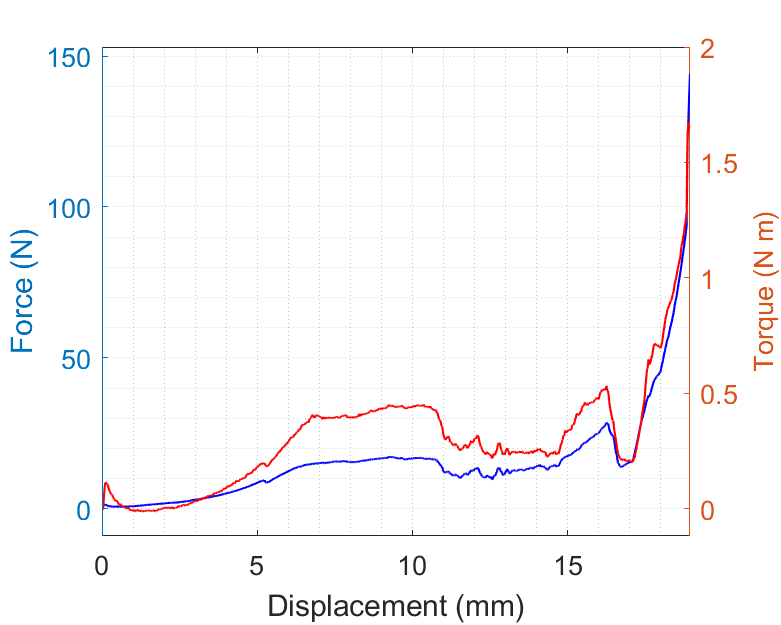

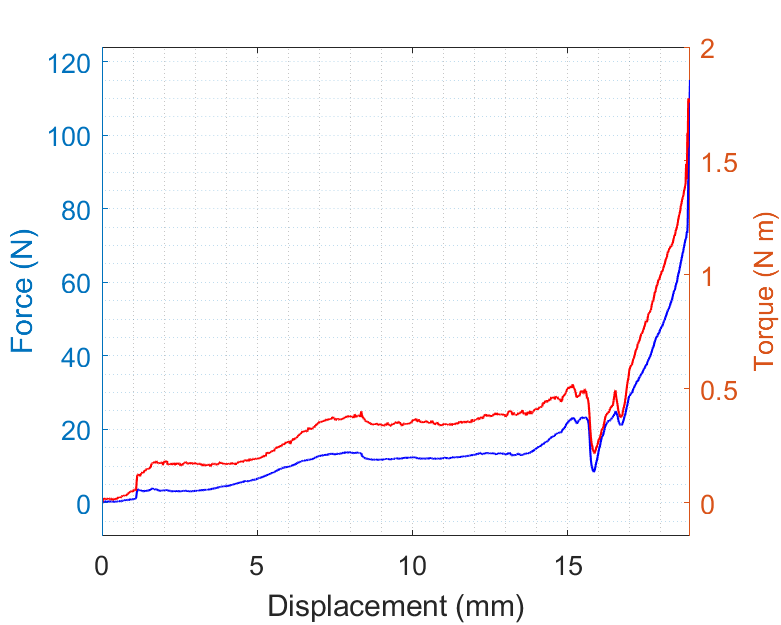


Figure 24. Sample 18 (left) sample 19 (right)


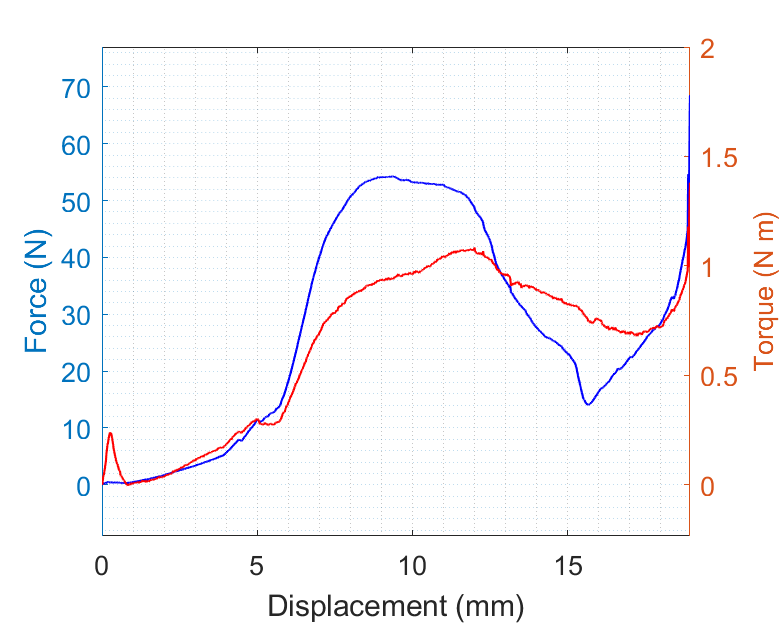

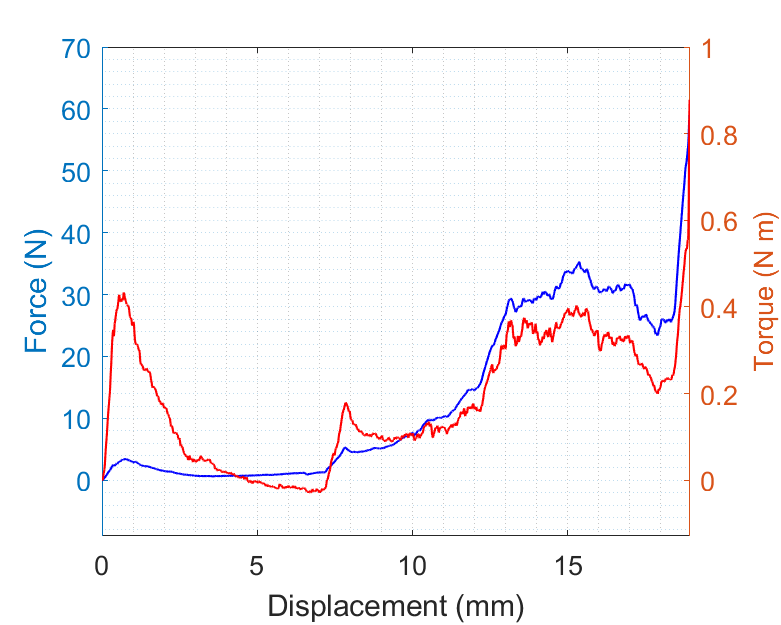


Figure 25. Sample 20 (left) sample 23 (right)


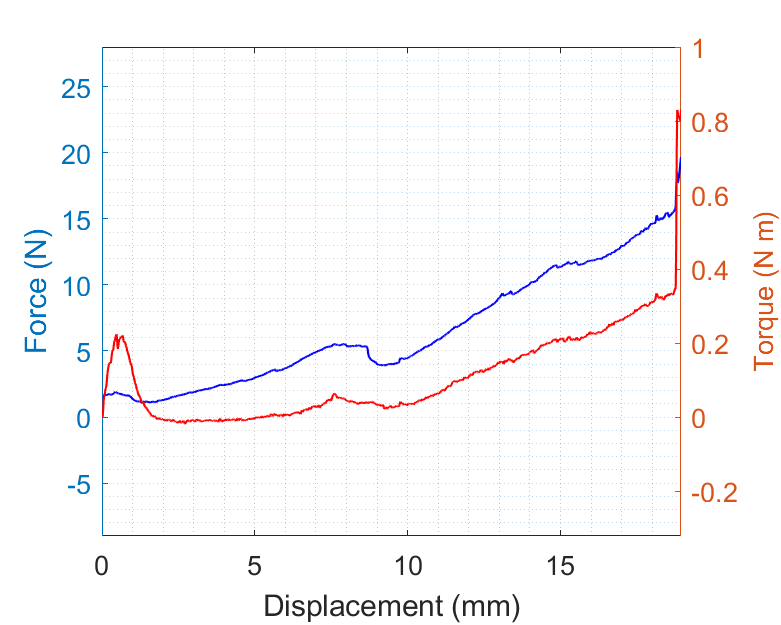


Figure 26. Sample 24

***Feed rate 0.03 mm/s***

*
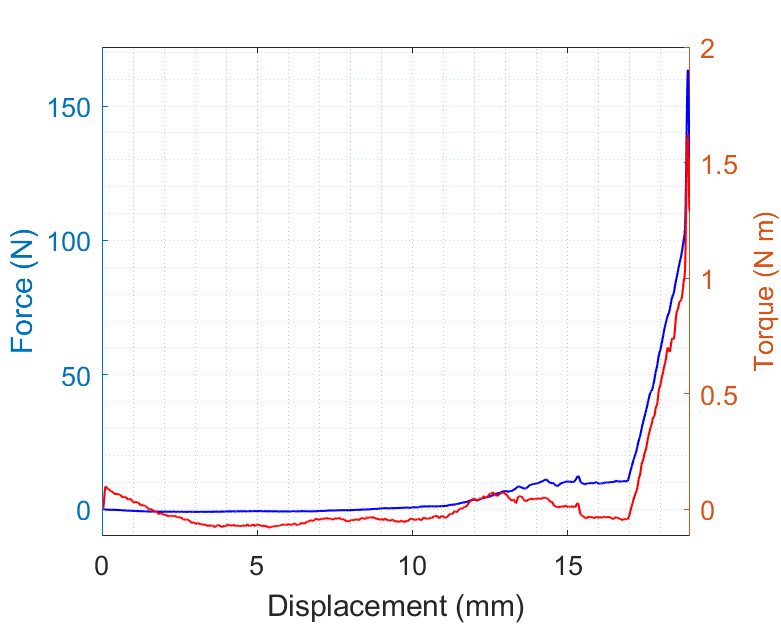
* ***
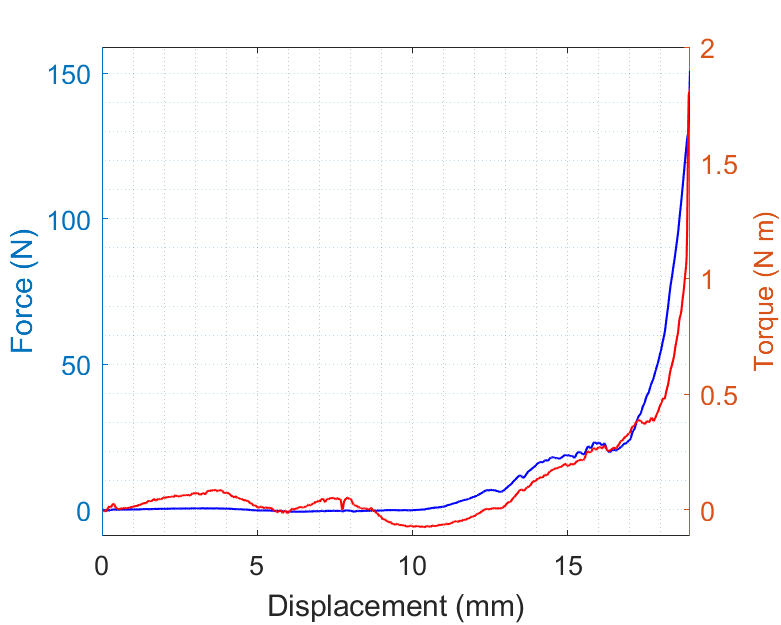
***

Figure 27. Sample 2 (left) and sample 3 (right)


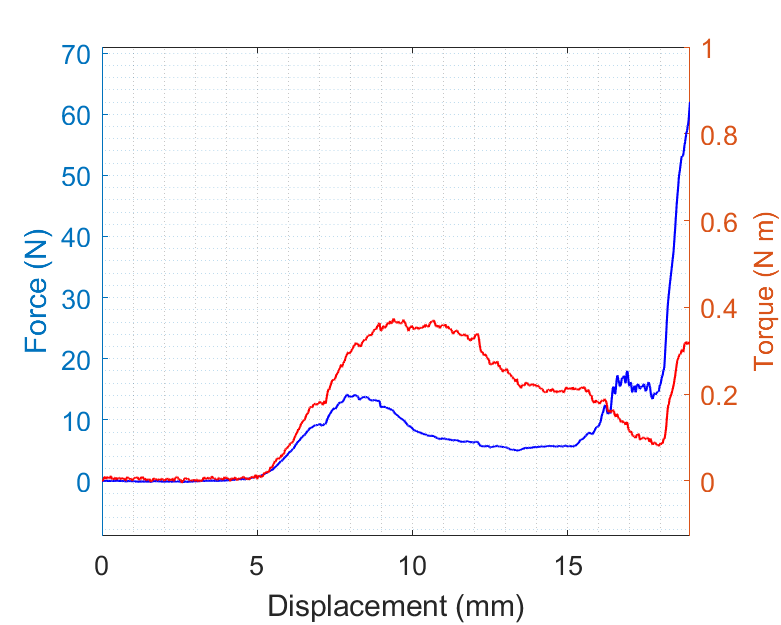

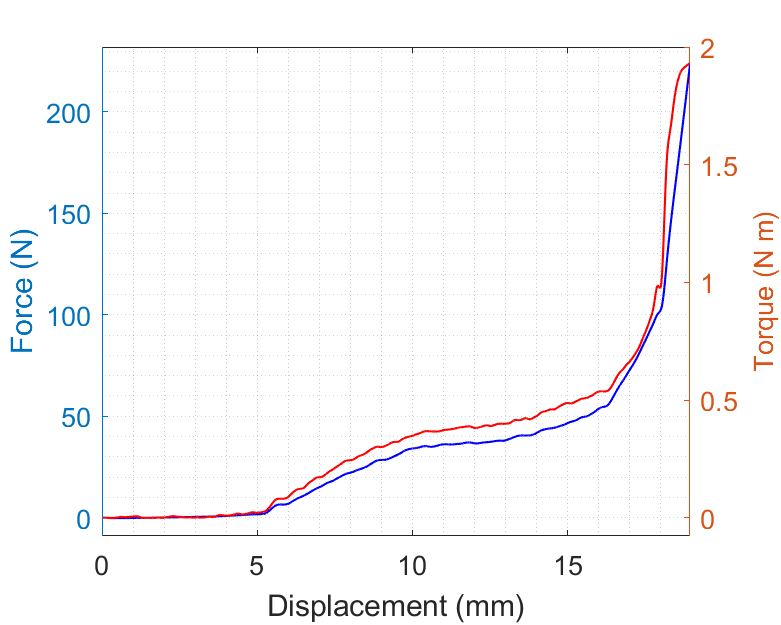


Figure 28. Sample 4 (left) and sample 5 (right)

*
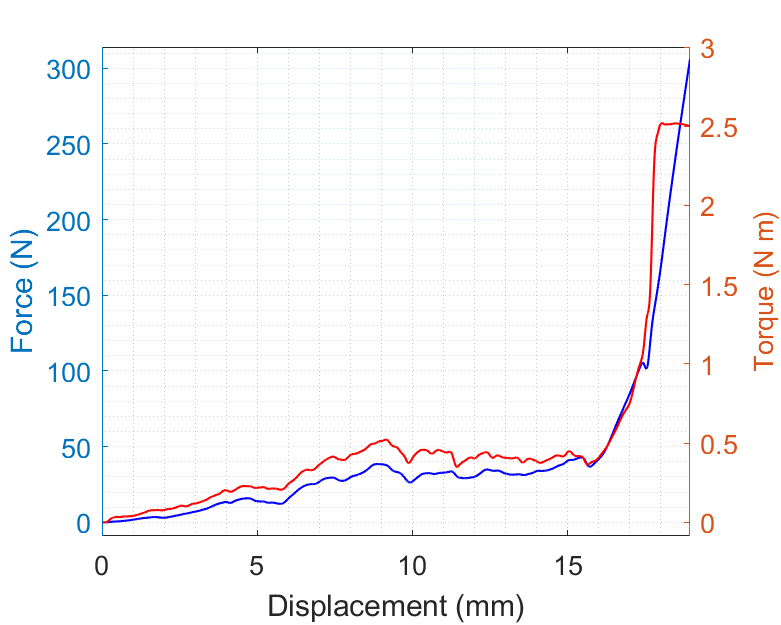
* *
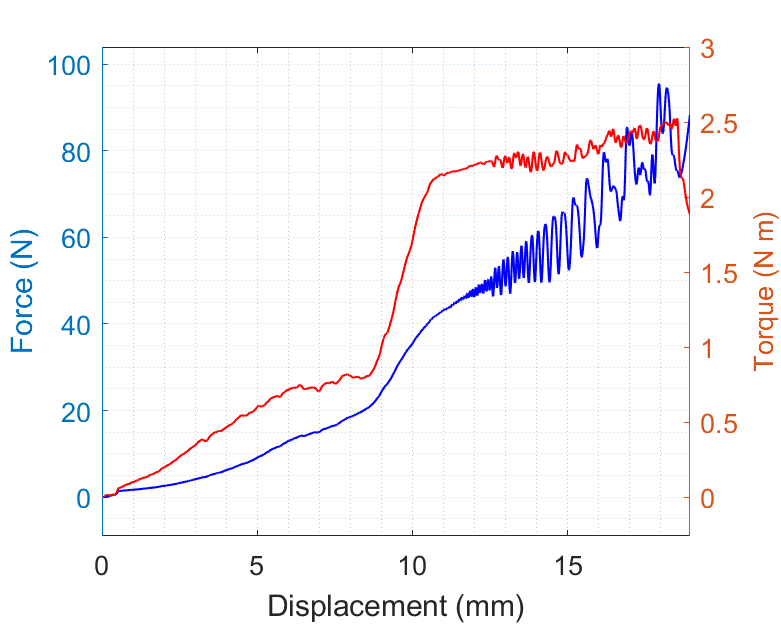
*

Figure 29. Sample 6 (left) and sample 7 (right)

*
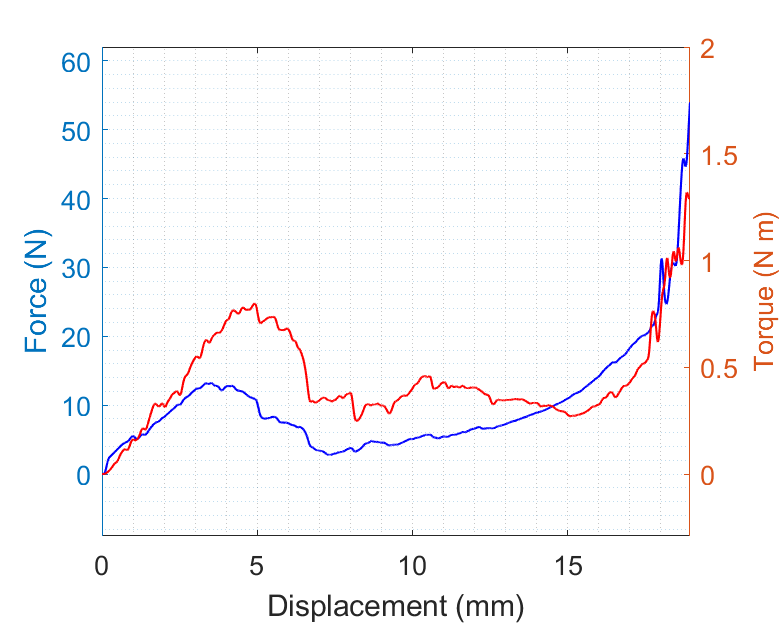
* *
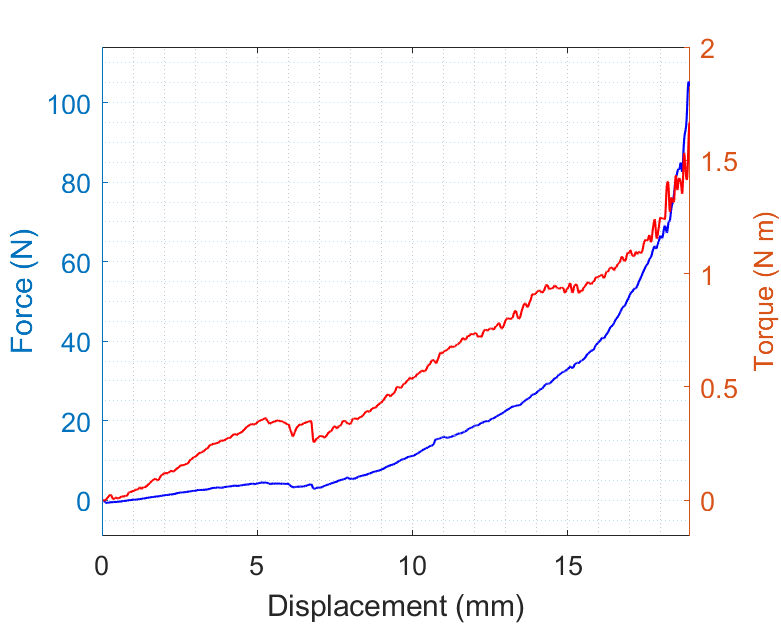
*

Figure 30. Sample 9 (left) and sample 10 (right)

*
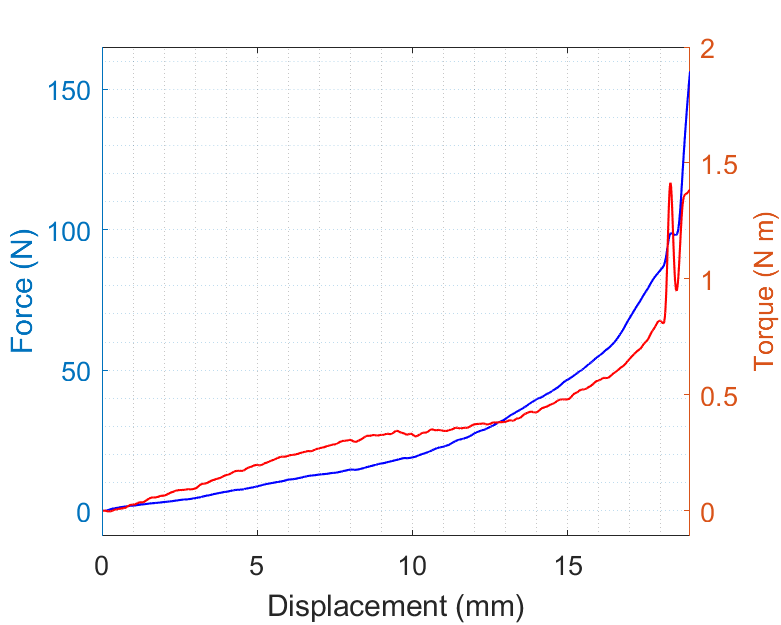
* *
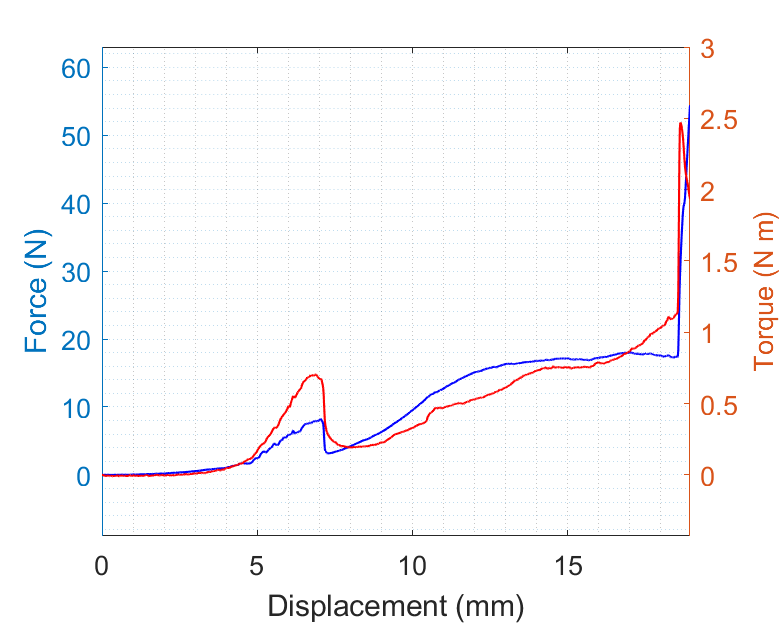
*

Figure 31. Sample 11 (left) and sample 12 (right)


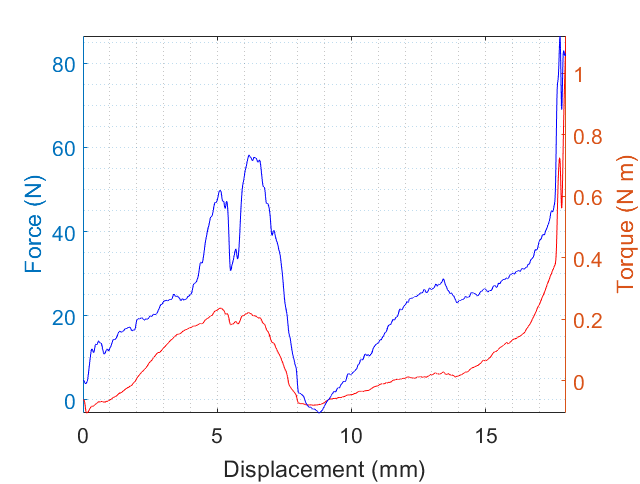


Figure 32. Sample 8
